# Efficacy and safety of rectal 5-aminosalicylic acid versus corticosteroids in active distal ulcerative colitis: a systematic review and network meta-analysis

**DOI:** 10.1038/srep46693

**Published:** 2017-04-25

**Authors:** Xiaojing Zhao, Changcheng Zhou, Jingjing Ma, Yunjuan Zhu, Min Sun, Peixue Wang, Yi Zhang, Haiqin Ma, Hongjie Zhang

**Affiliations:** 1 Department of Gastroenterology, First Affiliated Hospital of Nanjing Medical University, Nanjing, Jiangsu 210029, China; 2 Department of Urology, Nanjing First Hospital, Nanjing Medical University, 68 Changle Road, Nanjing, Jiangsu 210006, China; 3 Department of Oncology, Zhongnan Hospital of Wuhan University, Wuhan, Hubei 430071, China

## Abstract

Topical 5-aminosalicylic acid (5-ASA) and corticosteroids are used frequently in the treatment of active distal ulcerative colitis (UC). Our study aimed to determine the efficacy and safety of different topical drugs used to treat active distal UC. A random-effects model within a Bayesian framework was utilized to compare treatment effects and safety as odds ratios (ORs) with corresponding 95% credible intervals (CrI). The surface under the cumulative ranking area (SUCRA) and median rank (MR) with corresponding 95% CrI were calculated to rank the treatment outcomes. In the induction of clinical and endoscopic remission, most regimens showed significant advantages over placebo except topical budesonide 0.5 mg/d and hydrocortisone 100 mg/d. According to SUCRA and MR values, rectal 5-ASA 1.5 to 2.0 g/d + Beclomethasone dipropionate (BDP) 3 mg/d rendered the highest probability of being the best regimen to achieve clinical and endoscopic remission, followed by the separate use of 5-ASA 4 g/d and BDP 3 mg/d. The occurrence of adverse events was not significantly different between each treatments and placebo. In conclusion, the combined use of topical 5-ASA and BDP proved to be the best choice for active distal UC and further well-designed researches are warranted to assess its efficacy and safety.

Ulcerative colitis (UC) is characterized by diffuse and continuous inflammation of the colon. Currently, the etiology and pathogenesis remain unclear. According to a previous epidemiological study, approximately 75% of newly diagnosed UC patients have active distal UC^1^. Several studies showed that UC starts in the rectum and generally extends to the proximal colon^2^,^3^. A 5-year population-based follow-up study showed that among patients with proctitis, 28% had extension and 10% developed pancolitis^4^.

Until now, 5-ASA and corticosteroid preparations have proved to be the major therapeutic agents for the treatment of mild to moderate UC in clinical practice. The choice of appropriate regimens is dependent on the site of the disease and disease activity. Patients had limited distal colon inflammation (<60 cm maximum from the anal verge) can select topical preparations of 5-ASA or corticosteroids. For active distal UC, a rectal 5-ASA administration is recommended as the keystone and first-line therapy for inducing remission. Treatment with corticosteroids in UC patients was first investigated by Truelove and Wittsover 60 years ago^5^. However, conventional corticosteroid treatments are usually associated with systemic adverse events (AEs). Topical as well as second-generation corticosteroids (budesonide and beclomethasone) reduce the related systemic adverse-effects for the initially pass through hepatic metabolism and their potent anti-inflammatory effects^6^. Topical corticosteroids render a more favorable safety compared with conventional utilization and can be regarded as a suitable alternative to rectal 5-ASA. Several traditional meta-analyses have already been conducted to evaluate 5-ASA or corticosteroids for active distal UC^7^. However, these pair-wise meta-analyses were only limited to rectal 5-ASA or one specified agents of corticosteroids, and direct comparisons between each regimens were still unavailable. Thus, the optimal medication dose remains controversial.

Given the lack of head-to-head randomized controlled trials (RCTs) between 5-ASA and corticosteroids for active distal UC, we conducted a systematic review with network meta-analysis, which permitted the integration of direct and indirect evidences, and allowed us to compare the efficacy and safety of multiple regimens simultaneously.

## Methods

### Search strategies

We carried out a comprehensive electronic search of PUBMED, MEDLINE, EMBASE and the Cochrane Library, from inception to 15 May 2016, to identify all eligible studies. Both Medical Subject Heading (MeSH) terms and free words were used, including the following: “inflammatory bowel disease”, “IBD”, “Colitis, Ulcerative”, “ulcerative colitis”, “UC”, “Ulcerative Proctitis”, “UP” “Proctitis”, “Ulcerative proctosigmoiditis”, “UPS”, “Ulcerative sigmoiditis”, “enema”, “foam”, “suppositories”, “suppository”, “Administration, Topical”, “topical administration”, “Administration, Rectal” and “rectal administration”, “Mesalamine”, “mesalazine”, “5-aminosalicylic acid”, “5-ASA”, “5ASA”, “corticosteroids”, “steroids”, “glucocorticoids”, “budesonide”, “Beclomethasone dipropionate”, “BDP”, “prednisone”, “prednisolone”, “hydrocortisone”, “Betamethasone”. We also searched ClinicalTrials.gov website for eligible RCTs in progress. Meanwhile, previous systematic reviews and pair-wise meta-analysis were also explored to identify potential relevant studies. The search strategy involved no date or language restrictions.

### Selection criteria

Studies identified from the above-mentioned databases (PUBMED, MEDLINE, EMBASE and the Cochrane Library) were assessed by two independent authors according to the following inclusion criteria: (1) study design as RCTs; (2) trials enrolled active distal UC patients with disease margin <60 cm from the anal verge or distal to the splenic flexure (left-sided colitis, proctosigmoiditis and proctitis) at flexible colonoscopy or sigmoidoscopy; (3) trials comparing different doses of topical 5-ASA and corticosteroids with placebo or against each other. Additionally, dose-comparison studies of one agent were also eligible for inclusion; (4) drug utilization should be topical (i.e. enema, foam or suppository). Nevertheless, previous oral maintenance therapy, for instance 5-ASA class, could continue to be used during the trial; (5) studies included in our network-meta analysis necessarily reported the induction of clinical or endoscopic remission in active distal UC and the remission should be identified by a conventional or self-defined criteria; (6) duration of follow-up should not be less than 2 weeks.

The exclusion criteria were the following: (1) studies involved non-RCTs, reviews as well as meeting abstracts; (2) patients with a specific UC that spread beyond the splenic flexure or >60 cm from the anal verge; (3) we excluded studies in which topical drug utilization was just treated as a adjuvant treatment; (4) patients who presented with indeterminate colitis (IC), idiopathic proctitis or Crohn’s disease (CD) were excluded; and (5) studies that reported maintenance of remission in quiescent disease were also excluded.

### Data extraction

Two investigators extracted data from eligible studies independently, using a predesigned data-collection form. The primary outcomes were the clinical and endoscopic remission rates in active distal UC patients at 4 weeks (if the remission rate at 4 weeks was not available, the last time-point assessment in the trial would be taken). The secondary outcomes were induction of histopathological remission rates at 4 weeks (likewise, if the secondary outcomes at 4 weeks were not available, then they would be extracted from the end of the study) and the incidence of drug-related adverse events (AEs). Furthermore, the following information was extracted: last name of first author, publication year, the demographic characteristics of the patients, disease characteristics, interventions, concomitant therapy, duration of treatment and the scoring systems of remission. Any discrepancies between them were resolved by further discussion. If they did not reach a consensus, a third reviewer (HJZ) was consulted.

### Statistical analysis

Firstly, we performed traditional pair-wise meta-analyses for studies that directly compared different interventions using Stata software (version 12.0, StataCorp, College Station, TX, USA). To account for heterogeneity, the DerSimonian and Laird random effects model were used^8^. The heterogeneity between eligible studies was assessed with the *I*^2^ metric (25%, 50%, and 75% correspond to low, medium, and high levels of heterogeneity, respectively)^9^. We then performed a multiple-treatment network meta-analysis to combine the effect sizes of both direct and indirect comparisons with an extended random effects model proposed by Chaimani (obtained from www.mtm.uoi.gr) within a Bayesian framework. The posterior parameters were calculated by Markov chain Monte Carlo methods in the network meta-analysis^10^. Non-informative uniform and normal prior distributions were performed, and a randomly generated starting value was utilized to fit the model^11^. After an initial burn-in of 50,000, we conducted another 300,000 iterations. To rank treatments for each outcome, we calculated two metrics for each intervention: the median rank (MR) and the surface under the cumulative ranking area (SUCRA)^12^. Higher values of SUCRA suggested better efficacy and safety, whereas higher MR indicated a worse trend. All data syntheses in the network meta-analysis were undertaken using R (version 2.13.2, The R Foundation for Statistical Computing, www.r-project.org) and WinBUGS (version 1.4.3, MRC Biostatistics Unit, Cambridge, UK) with the R2WinBUGS package (version 2.1-21, cran.r-project.org/web/packages/R2WinBUGS). For dichotomous variables treatment effects were summarized as odds ratios (ORs) with their corresponding 95% confidence intervals (CI) or credible intervals (CrI) (CI for direct evidence, and CrI for indirect evidence or network evidence), respectively.

The consistency of the network, defined as the discrepancy of results derived from direct and indirect comparisons, was assessed by inconsistency factors and their 95% CI in closed loops (loops in which their CI did not contain zero were regarded as statistical inconsistencies)^13^. Nevertheless, we also compared the pooled ORs from network meta-analysis and traditional pair-wise meta-analysis to further verify the consistency of the network. The goodness of fit of the model was examined by calculating the posterior mean residual deviance, and the model was considered to fit the data well when the posterior mean residual deviance approximated the number of data points in the present study^14^.

To detect the small study effects on the data, we conducted comparison-adjusted funnel plots^13^. Sensitivity analysis was conducted to authenticate the robustness of our analyses according to the quality of included studies (excluding studies with a high risk of bias). This study was conducted and reported in accordance with the PRISMA guidelines^15^.

### Assessment of risk of bias

The Cochrane Collaboration tool was used to assess the methodological quality of included studies^16^. It addressed the following items: random sequence generation, allocation concealment, blinding of patients, personnel and outcome assessment, incomplete outcome data, selective reporting, and other sources of bias.

### Quality of evidence

The quality of the therapeutic effect for primary outcomes (clinical and endoscopic remission) was estimated using a four-step approach based on the Grading of Recommendations Assessment, Development and Evaluation (GRADE) system^17^. Evidence evaluation included direct, indirect and network estimates. The quality of evidence was rated as high, moderate, low and very low. At the beginning of the assessment, the quality of direct evidence was considered high, but could be rated down for the following reasons: (i) risk of bias; (ii) inconsistency; (iii) indirectness; (iv) imprecision; and (v) publication bias. The rating for indirect evidence from the lower rating of the quality of direct evidence would be further rated down because of imprecision and indirectness. Finally, the higher rating of direct and indirect evidence was used as the quality rating for the network estimates.

## Results

### Characteristics of included studies

**Figure 1:**
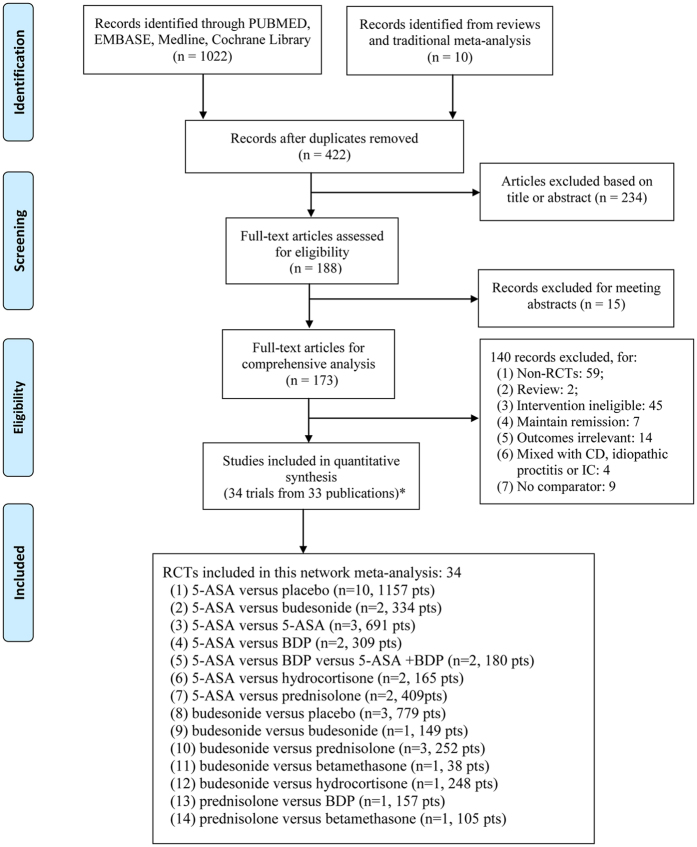
Flow diagram. Thirty-three articles containing 34 studies were included in this network meta-analysis. RCT. randomised controlled trial. CD. Crohn’s disease; IC. indeterminate colitis; 5-ASA, 5-aminosalicylic acid; BDP, beclomethasone dipropionate; pts, patients. *One publication reported two trials.

The flow diagram of the study selection is summarized in Fig. 1. Firstly, 1032 studies were identified in our initial research. After removal of duplicates, 422 citations remained. Then, 234 citations were excluded based on the title or abstract and we further scanned full-texts for the remaining 188 articles. Fifteen trials were excluded for meeting abstracts, and 140 studies were further removed for: non-RCTs, reviews, intervention ineligible, maintain remission, outcomes irrelevant, mixed with CD, idiopathic proctitis or IC, and absence of comparator. Finally, 33 articles reporting 34 eligible RCTs, which enrolled a total of 4973 subjects, were included in the network meta-analysis.

The baseline characteristics of the included studies are shown in Table 1. The 34 eligible RCTs (31 for clinical remission, 23 for endoscopic remission) included two-arm (n = 28)^18^,^19^,^20^,^21^,^22^,^23^,^24^,^25^,^26^,^27^,^28^,^29^,^30^,^31^,^32^,^33^,^34^,^35^,^36^,^37^,^38^,^39^,^40^,^41^,^42^,^43^,^44^, three-arm (n = 3)^45^,^46^,^47^ and four-arm (n = 3)^48^,^49^,^50^ RCTs, which enrolled 4973 active distal UC patients. Nineteen eligible RCTs compared 5-ASA(n = 11)^19^,^20^,^29^,^36^,^37^,^38^,^39^,^44^,^47^,^49^,^50^ as well as budesonide (n = 3)^18^,^48^ with placebo or against each other (n = 2)^21^,^32^, or different doses of 5-ASA(n = 2)^22^,^27^ and budesonide (n = 1)^28^. Fifteen eligible RCTs compared 5-ASA with BDP (n = 2)^23^,^24^, 5-ASA + BDP (n = 2)^45^,^46^, prednisolone (n = 2)^31^,^41^ and hydrocortisone (n = 2)^33^,^42^, or budesonide with prednisolone (n = 3)^34^,^35^,^40^. Additionally, four comparisons concerning budesonide, betamethasone, hydrocortisone, prednisolone and BDP were investigated by four RCTs^25^,^26^,^30^,^43^.

### Risk of bias in included studies

The risk of bias in all included studies is shown in Supplementary Figure S1. Twenty-five studies provided sufficient details of randomization. Seventeen studies were inadequate in terms of allocation concealment. One study was not blind to participants or study personnel and one trial had a high risk of bias because of unblinded outcomes. Considering the incomplete outcome data domain, twenty-four studies were cited as a having a low risk of bias. Seventeen studies had a low risk of reporting bias.

## Primary Outcome

### Efficacy of rectal 5-ASA and corticosteroids for the induction of clinical remission in active distal UC patients

**Figure 2:**
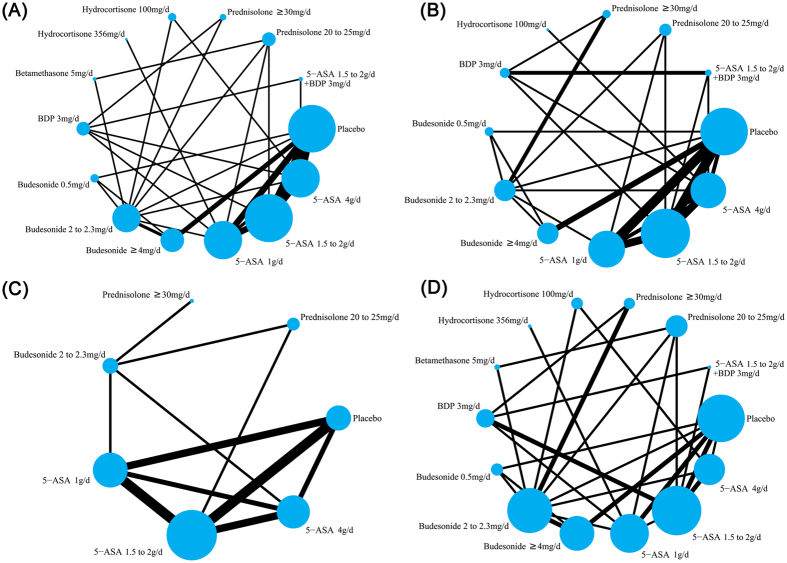
Network of eligible randomised controlled trials (RCTs) for comparisons of efficacy and safety between rectal mesalazine, corticosteroids and placebo. The thickness of the connecting lines represents the number of trials between each comparator, and the size of each node corresponds to the number of subjects who received the same pharmacological agent (sample size). (**A**) Clinical remission. (**B**) Endoscopic remission. (**C**) Histopathological remission. (**D**) Adverse events. 5-ASA, 5-aminosalicylic acid; BDP, beclomethasone dipropionate.

Comparisons of the induction of clinical remission with various medical therapies in active distal UC patients by network meta-analysis are shown in Fig. 2(A). Thirty-one eligible RCTs enrolled 4724 active distal UC patients were included in the study to assess the induction of clinical remission. Among them, twenty-six two-arm, two three-arm and three four-arm RCTs compared 5-ASA or corticosteroids (e.g. budesonide, BDP, betamethasone, hydrocortisone, prednisolone) with placebo or against each other. In total, 4724 patients with distal UC were assigned to 5-ASA (n = 1969), budesonide (n = 986), BDP (n = 281), betamethasone (n = 67) prednisolone (n = 437), hydrocortisone (n = 208), 5-ASA + BDP (n = 40) and placebo group (n = 736).

In pair-wise meta-analysis, all treatments exerted a trend of improvement in clinical remission when compared with placebo, however, only rectal 5-ASA 1 g/d, or higher dosage (1.5 to 2.0 and 4 g/d) showed statistical significance compared with placebo (OR 6.22, 95% CI: 3.86–10.01; OR 7.11, 95% CI: 3.48–14.52 and OR 5.62, 95% CI: 3.28–9.65, respectively), as well as budesonide ≥4 mg/d and 2 to 2.3 mg/d (OR 2.72, 95% CI: 1.86–3.99 and OR 2.79, 95% CI: 1.22–6.37) (Table 2). The results of comparisons on induction of clinical remission in our network meta-analysis are shown in Table 3(A). As a combination therapy, 5-ASA 1.5 to 2.0 g/d + BDP 3 mg/d demonstrated significant superiority over placebo (OR 29.22, 95% CrI: 5.15–117.49). Additionally, 5-ASA 4 g/d (OR 6.35, 95% CrI: 4.33–9.26), 5-ASA1.5 to 2.0 g/d (OR 6.30, 95% CrI: 4.33–9.08), 5-ASA 1 g/d (OR 5.57, 95% CrI: 3.70–8.23), budesonide ≥4 mg/d (OR 2.88, 95% CrI: 1.99–4.26), budesonide 2 to 2.3 mg/d (OR 2.30, 95% CrI: 1.50–3.47), BDP 3 mg/d (OR 6.69, 95% CrI: 3.78–11.54), betamethasone 5 mg/d (OR 6.52, 95% CrI: 2.47–14.58), hydrocortisone 356 mg/d (OR 4.60, 95% CrI: 1.31–12.06), prednisolone ≥30 mg/d (OR 6.38, 95% CrI: 2.65–13.08) and prednisolone 20 to 25 mg/d (OR 2.88, 95% CrI: 1.68–4.84) also showed significant superiority over placebo for the rate of clinical remission.

**Figure 3:**
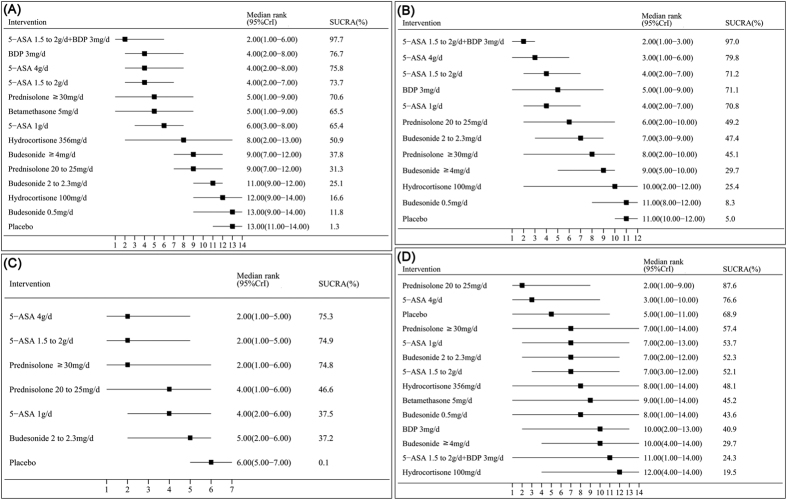
Surface under the Cumulative Ranking Curve (SUCRA), expressed as percentages, ranking the therapeutic effects and safety of treatments for active distal ulcerative colitis patients. For efficacy and safety assessment, the pharmacological agent with the highest SUCRA value would be the most efficacious and safe treatment. (**A**) Clinical remission. (**B**) Endoscopic remission. (**C**) Histopathological remission. (**D**) Adverse events. 5-ASA, 5-aminosalicylic acid; BDP, beclomethasone dipropionate.

As depicted in Fig. 3(A), we estimated the ranking probability via SUCRA and MR, which indicated that 5-ASA 1.5 to 2.0 g/d + BDP 3 mg/d (SUCRA = 97.7%; MR 2.00, 95% CI: 1.00–6.00) had the highest probability of being the best treatment to achieve clinical remission, followed by BDP 3 mg/d (SUCRA = 76.7%; MR 4.00, 95% CI: 2.00–8.00) and 5-ASA 4 g/d (SUCRA = 75.8%; MR 4.00, 95% CI: 2.00–8.00).

### Efficacy of rectal 5-ASA and corticosteroids to induce endoscopic remission in active distal UC patients

Comparisons of the induction of endoscopic remission with various medical therapies in active distal UC patients are shown in Fig. 2(B). Twenty-three eligible RCTs enrolled 3469 active distal UC patients were included in our network meta-analysis for the induction of endoscopic remission. Among them, seventeen two-arm, three three-arm and three four-arm eligible studies compared different doses of 5-ASA or corticosteroids (e.g. budesonide, BDP, hydrocortisone, prednisolone) with placebo or against each other.

Pair-wise meta-analysis, as shown in Table 2, indicated that rectal 5-ASA 1 g/d, or higher dosage (1.5 to 2.0 and 4 g/d) had significant superiority over placebo in inducing endoscopic remission (OR 6.45, 95% CI: 4.23–9.82; OR 4.49, 95% CI: 2.61–7.73 and OR 6.86, 95% CI: 3.53–13.34, respectively). Besides, rectal budesonide ≥4 mg/d and budesonide 2 to 2.3 mg/d could also significantly improve the endoscopic remission in active distal UC patients when compared with placebo (OR 2.29, 95% CI: 1.42–3.71 and OR 3.15, 95% CI: 1.29–7.70). The pooled results of network meta-analysis for the induction of endoscopic remission are shown in Table 3(B). As a combination therapy, 5-ASA 1.5 to 2.0 g/d + BDP 3 mg/d demonstrated significantly superiority over the placebo (OR 17.00, 95% CrI: 5.21–41.18). Not only the combined regimen, the separate rectal use of 5-ASA 4 g/d (OR 5.36, 95% CrI: 3.26–8.38), 5-ASA 1.5 to 2.0 g/d (OR 4.89, 95% CrI: 3.22–7.16), 5-ASA 1 g/d (OR 4.97, 95% CrI: 3.21–7.51) rectal budesonide ≥4 mg/d (OR 2.55, 95% CrI: 1.55–4.12), budesonide 2 to 2.3 mg/d (OR 3.53, 95% CrI: 1.90–5.95), BDP 3 mg/d (OR 4.66, 95% CrI: 2.21–8.67), prednisolone ≥30 mg/d (OR 3.25, 95% CrI: 1.35–6.73) and prednisolone 20 to 25 mg/d (OR 3.76, 95% CrI: 1.92–6.76) also rendered significant superiority over placebo for the induction of endoscopic remission.

As depicted in Fig. 3(B), we estimated the ranking probability via SUCRA and MR, which indicated that 5-ASA 1.5 to 2.0 g/d + BDP 3 mg/d (SUCRA = 97.0%; MR 2.00, 95%CI: 1.00–3.00) had the highest probability of being the best treatment to achieve endoscopic remission, followed by 5-ASA4g/d (SUCRA = 79.8%; MR 3.00, 95%CI: 1.00–6.00) and 5-ASA1.5 to 2.0 g/d (SUCRA = 71.2%; MR 4.00, 95%CI: 2.00–7.00).

## Secondary outcome

### Efficacy of rectal 5-ASA and corticosteroids for induction of histopathological remission in active distal UC patients

Comparisons of the induction of histopathological remission with various medical therapies in active distal UC patients are shown in Fig. 2(C). Ten eligible studies enrolled 1776 active distal UC patients were included in our network meta-analysis for the induction of histopathological remission. Among them, seven two-arm, one three-arm and two four-arm eligible studies compared rectal 5-ASA or corticosteroids (budesonide and prednisolone) with placebo or against each other.

Results from direct evidences indicated that rectal 5-ASA 1 g/d, or higher dosage (1.5 to 2.0 and 4 g/d) could obviously increase the histopathological remission rate when compared with placebo (OR 4.36, 95% CI: 1.82–10.42; OR 5.75, 95% CI: 2.11–15.70 and OR 9.49, 95% CI: 2.14–42.04, respectively) (Table 2). The pooled results derived from network meta-analysis regarding the rate of histopathological remission are shown in Table 3(C). 5-ASA 4 g/d (OR 8.64, 95% CrI: 3.68–20.26), 5-ASA 1.5 to 2.0 g/d (OR 8.56, 95% CrI: 3.79–17.29), 5-ASA 1 g/d (OR 6.33, 95% CrI: 2.67–13.32) had obvious advantages than placebo for the induction of histopathological remission. Likewise, budesonide 2 to 2.3 mg/d (OR 5.75, 95% CrI: 1.87–13.60), prednisolone ≥30 mg/d (OR 14.68, 95% CrI: 1.71–62.94) and prednisolone 20 to 25 mg/d (OR 6.82, 95% CrI: 1.93–18.55) also showed significant superiorities over placebo.

The estimated SUCRA and MR indicated that 5-ASA 4 g/d (SUCRA = 75.3%; MR 2.00, 95% CI: 1.00–5.00) had the highest probability of being the best treatment for inducing histopathological remission, followed by 5-ASA 1.5 to 2.0 g/d (SUCRA = 74.9%; MR 2.00, 95% CI: 1.00–5.00) and prednisolone ≥30 mg/d (SUCRA = 74.8%; MR 2.00, 95% CI: 1.00–6.00).

### Safety and Adverse events (AEs)

Comparisons of the incidence of AEs in our network are shown in Fig. 2(D). Twenty-eight eligible studies enrolled 4077 active distal UC patients were included in our network meta-analysis for the incidence of AEs. Among them, twenty-five two-arm, two three-arm and one four-arm eligible studies compared 5-ASA or corticosteroids with placebo or against each other.

The pair-wise meta-analysis regarding AEs showed no statistically significant difference for almost all regimens compared with placebo (Table 2). The occurrence of AEs yielded from network meta-analysis also demonstrated no statistical difference between each regimens and placebo Table 3(D).

The corresponding SUCRA and MR values are shown in Fig. 3(D). According to the SUCRA and MR, prednisolone 20 to 25 mg/d (SUCRA = 87.6%; MR 2.00, 95%CI: 1.00–9.00) had the highest probability of being the safest treatment for active distal UC patients, followed by 5-ASA 4 g/d (SUCRA = 76.6%; MR 3.00, 95%CI: 1.00–10.00) and placebo (SUCRA = 68.9%; MR 5.00, 95%CI: 1.00–11.00). However, hydrocortisone 100 mg/d ranked the worst (SUCRA = 19.5%; MR 12.00, 95%CI: 4.00–14.00).

### Evaluation of consistency and fit of the models

The results of the pair-wise and corresponding Bayesian network meta-analysis are shown in Table 2. The effect size and relevant CI or CrI delivered no obvious discrepancy between the two different types of comparisons, indicating that there were no inconsistencies. Moreover, the consistency was also confirmed by the quantitative assessment in closed loops (Supplementary Figure S2). The result of the model test indicated that the posterior mean residual deviance approximated the data points in both the primary and secondary outcomes (Table 4); namely, the present model fitted the data well.

### Quality of evidence

The GRADE approach was applied to the primary outcomes of clinical and endoscopic remission (Supplementary Tables S1 and S2). The quality of direct and indirect evidence was very low, or low or moderate for all comparisons. Unfortunately, the quality of network meta-analysis was also not satisfactory.

### Publication bias and sensitivity analysis

The result of the comparison-adjusted funnel plots did not reveal any evidence of apparent asymmetry (Supplementary Figure S3). Sensitivity analysis regarding the quality of the study did not significantly alter the results of the two outcomes (Supplementary Table S3).

## Discussion

In this network meta-analysis, we included 34 RCTs comparing the efficacy (31 for clinical remission, 23 for endoscopic remission and 10 for histopathological remission) and safety (28 for incidence of AEs) of different treatment strategies in patients with active distal UC, and provided some hierarchies of agents for clinicians in the treatment process. The results showed that topical 5-ASA 1.5 to 2.0 g/d + BDP 3 mg/d rendered the highest probability of being the best regimen to induce clinical and endoscopic remission in active distal UC patients compared with placebo.

We obtained certain important conclusions from this network meta-analysis. First, the efficacy and safety of 5-ASA were consistent with the guidelines for UC clinical practice published by the American College of Gastroenterology^51^, which suggested that topical 5-ASA at different doses (1–4 g) are efficacious to induce remission in active distal UC. Similarly, a previous meta-analysis^52^ concluded that topical 5-ASA is an effective first-line treatment for patients with left-sided colitis and ulcerative proctitis. Our network meta-analysis demonstrated that topical 5-ASA at different doses (1–4 g/day) showed significant advantages over placebo in inducing clinical, endoscopic and histopathological remission and also did not increase the rate of AEs. Besides, 5-ASA 4 g/d was further demonstrated to be no significant superiority over the 5-ASA 1.5 to 2 g/d and 5-ASA 1 g/d regimens, which was consistent with the previous studies^49^,^50^. However, it was worth noting that 5-ASA 4 g/d had a higher probability of being the best choice for inducing active distal UC remission than 5-ASA at 1.5 to 2 and 1 g/d. Among the patients enrolled in the 34 eligible RCTs, 1969 subjects were assigned to 5-ASA therapy. Large cohorts in our study might have sufficiently powerful effect sizes to show statistical differences between the treatment and placebo groups and have sufficient credibility to validate our conclusion.

Additionally, our results derived from network meta-analysis also suggested that among all regimens, 5-ASA 1.5 to 2.0 g/d + BDP 3 mg/d had the highest probability of being the best treatment to induce clinical and endoscopic remission. However, only two RCTs including 59 patients were assigned to 5-ASA 1.5 to 2.0 g/d + BDP 3 mg/d group in the network meta-analysis. Small cohorts in this study might not provide sufficient power to support our conclusion. Moreover, the absence of studies that reported histopathological remission induced by topical combination regimen restricted a further detection of the combined efficacy. Consequently, the certain effect of topical combination regimen should be further authenticated by well-designed RCTs with comprehensive end-points.

Topical administration of corticosteroids also showed advantages over placebo in inducing clinical and endoscopic remission, except topical budesonide 0.5 mg/d and hydrocortisone 100 mg/d. Unfortunately, we failed to assess most of their effects on histopathological remission for the remission rate was unavailable. A study conducted by Hanauer *et al*.^48^ confirmed the presence of dose-related effectiveness of topical budesonide compared with the placebo. The present study also demonstrated that budesonide 0.5 mg/d showed no significant advantages over placebo in inducing clinical and endoscopic remissions for active distal UC, whereas a higher dose rendered it more efficient.

In safety assessment, most regimens had a trend to cause more AEs than placebo, but no significant differences were identified. SUCRA values demonstrated that prednisolone at 20 to 25 mg/d would be the most safe treatment for active distal UC patients, followed by 5-ASA 4 g/d and placebo. However, the regimen of rectal hydrocortisone 100 mg/d might have the highest probability to increase AEs in active distal UC patients. For the varied definitions of AEs and selected reports of serious AEs and AE-related withdrawals in the included RCTs, the predicted results should be further confirmed. Nevertheless, this ranked results suggested that hydrocortisone 100 mg/d should be applied cautiously in clinical practice.

Our study had certain strengths. First, it is the first network meta-analysis to provide comprehensive comparisons on available interventions for patients with active distal UC. Second, we introduced a rank order of the various regimens included in our study to provide some hierarchies for physicians in clinical practice. Finally, we applied the latest guidelines of the GRADE approach to evaluate the quality of evidences for the primary outcomes. However, our network meta-analysis has several limitations. First, only different doses and durations of 5-ASA and corticosteroids were taken into consideration in the study, and the influence of formulation was unable to further detect. Second, the definitions of clinical, endoscopic and histological remissions as well as AEs were varied in the included trials, which could lead to a potential bias. Third, trials included in the present study seldom reported the outcome of histological remission, which limited the comprehensive assessment of efficacy. Finally, most network evidences delivered low qualities, based on the GRADE assessment.

In conclusion, our network meta-analysis showed that the combination of 5-ASA 1.5 to 2.0 g/d and BDP 3 mg/d had the highest probability of being the best treatment to induce clinical and endoscopic remission in active distal UC patients among all treatment strategies, followed by the separate use of 5-ASA 4 g/d and BDP 3 mg/d. In the future, additional high quality RCTs are warranted to further assess the efficacy and safety of topical 5-ASA and corticosteroids.

## Additional Information

**How to cite this article**: Zhao, X. *et al*. Efficacy and safety of rectal 5-aminosalicylic acid versus corticosteroids in active distal ulcerative colitis: a systematic review and network meta-analysis. *Sci. Rep.* **7**, 46693; doi: 10.1038/srep46693 (2017).

**Publisher's note:** Springer Nature remains neutral with regard to jurisdictional claims in published maps and institutional affiliations.

## Supplementary Material

Supplementary Information

## Figures and Tables

**Table 1:** Characteristics of the included studies.

Author (year)	Country	No. of patients	Mean age	Patients	Interventions	Concomitant therapy
Sandborn (2015)	USA	134/147	44.3/41.9	mild to severe UP or UPS	budesonide foam 4 mg/day for 2 weeks, then 2 mg/day for 4 weeks vs placebo	5-ASA
Sandborn (2015*)	USA	133/132	43.2/41.4	mild to moderate UP or UPS	budesonide foam 4 mg/day for 2 weeks, then 2 mg/day for 4 weeks vs placebo	5-ASA
Crispino (2015)	Italy	40/40/40	52/54/53	mild to moderately active distal UC	5-ASA 4 g/day vs BDP 3 mg/day vs 5-ASA 1.5 g + BDP 3 mg/day enema	5-ASA
Kobayashi (2014)	Japan	64/61	—	mild to moderately active distal UC	5-ASA suppository 1 g vs placebo	5-ASA
Watanabe (2013)	Japan	65/64	41.9/41.3	mild to moderate UP	5-ASA suppository 1 g vs placebo	5-ASA or sulfasalazine
Hartmann (2010)	Germany	118/119	41.8/43.6	mild to moderate left-sided UC	budesonide enema 2 mg/day vs 5-ASA enema 4 g/day	5-ASA
Andus (2010)	Multicenter	200/203	41.4/42.7	mild to moderately active UP	5-ASA suppository 1 g/day vs 5-ASA suppository 1.5 g/day	No
Biancone (2007)	Italy	50/42	—	mild to moderate distal UC	BDP enema 3 mg/day vs 5-ASA enema 2 g/day	5-ASA or sulfasalazine
Gionchetti (2005)	Italy	111/106	42/41	mild to moderate active left-sided UC UP, and UPS	BDP enema 3 mg/day vs 5-ASA enema 1 g/day	5-ASA or sulfasalazine
Hammond (2004)	Germany	22/16	43.1/42.4	active distal UC	budesonide foam 2 mg/day vs betamethasone(BMT) enema 5 mg bid for the first 2 weeks and OD for the next fortnight	5-ASA or sulfasalazine
Bar-Meir (2003)	Multicenter	120/128	42/42	active UP or UPS	budesonide foam 2 mg/day vs hydrocortisone acetate foam100 mg/day	5-ASA
Malchow (2002)	Multicenter	111/118	—	active distal UC	5-ASA foam 2 g/day vs 5-ASA enema 4 g/day	5-ASA or sulfasalazine
Lindgren (2002)	Sweden	73/76	—	active distal UC or UP	budesonide enema 2 mg/day vs budesonide enema 4 mg/day	No
Pokrotnieks(2000)	Multicenter	54/57	44.1/45.4	mildly to moderately active UP, UPS, or left-sided UC	5-ASA foam 2 g/day vs placebo	5-ASA, olsalazine, or sulfasalazine
Franzè (1999)	Italy	30/29	—	active distal UC	BDP enema 3 mg/day vs 5-ASA enema 4 g/day	—
Hanauer (1998)	USA	60/57/56/60	43/39/42/40	active distal UC	placebo enema vs budesonide 0.5, 2, or 8 mg enema	5-ASA
Hanauer (1998*)	USA	73/71/73/70	40.7/42.4/37.7/39.5	active distal UC	5-ASA enema 1, 2, or 4 g/day vs placebo	5-ASA
Campieri (1998)	Italy	80/77	41.2/42.2	active distal UC	BDP enema 3 mg/day vs prednisolone enema 30 mg/day	5-ASA or sulfasalazine
Mulder (1996)	Netherlands	19/20/21	36.3/39.8/42.95	active UP or UPS	BDP 3 mg + 5-ASA 2 g enema vs BDP 3 mg enema vs 5-ASA 2 g enema	5-ASA or sulfasalazine
Lee (1996)	UK	149/146	44/45	active distal UC	5-ASA foam 2 g/day vs prednisolone foam 20 mg/day	5-ASA or sulfasalazine
Lemann (1995)	Multicenter	48/49	39/38	active distal UC or UP	budesonide enema 2 mg/day vs 5-ASA enema 1 g/day	5-ASA
Lofberg (1994)	Multicenter	45/55	41/38	active distal UC	budesonide enema 2 mg/day vs prednisolone enema 25 mg/day	5-ASA, olsalazine, or sulfasalazine
Porro (1994)	Italy	44/44	42.6/43.3	active distal UC or UPS	budesonide enema 2.0 mg/day vs prednisolone enema 20 mg/day	5-ASA or sulfasalazine
Farup (1994)	Norwegian	41/38	40/39	active UP and UPS	5-ASA suppositories 1 g/day vs hydrocortisone foam 356 mg/day	5-ASA or sulfasalazine
Campieri (1991)	Italy	27/30/29/27	36/42/37/40	mild to moderate distal UC	5-ASA enema 1, 2, or 4 g/day vs placebo	sulphasalazine
Campieri (1990)	Italy	32/30	37/34	mild to moderate distal UC	5-ASA suppositories 1.5 g/day vs placebo	sulphasalazine
Campieri (1990*)	Italy	32/31/31	42.1/37.1/41.2	mild to moderate UP or UPS	5-ASA suppositories 1 g/day vs 5-ASA suppositories 1.5 g/day vs placebo	5-ASA or sulfasalazine
Danielsson (1987)	Sweden	31/33	—	active distal UC	budesonide 2 mg enema vs prednisolone 25 mg enema	5-ASA or sulfasalazine
Williams (1987)	Canada	13/11	37.3/42.7	active UP	5-ASA suppositories 1.5 g/day vs placebo	sulfasalazine or prednisone
Sutherland (1987)	Canada	29/30	40/36	active distal UC	5-ASA enema 4 g/day vs placebo	sulfasalazine or prednisone
Sutherland(1987*)	Multicenter	76/77	40/38	active distal UC	5-ASA enema 4 g/day vs placebo	sulfasalazine or prednisone
Binder (1987)	Denmark	53/61	36/40.1	mild to moderate UP or UPS	5-ASA enema 1 g/day vs prednisolone enema 25 mg/day	sulphasalazine
Campieri (1981)	Italy	44/42	40/37	mild to moderate distal UC	5-ASA enema 4 g/day vs hydrocortisone enema 100 mg/day	sulphasalazine
Jones (1971)	UK	51/54	41.5/41	active UP or left-sided UC	betamethasone enema 5 mg/day vs prednisolone enema 20 mg/day	sulphasalazine
**Author (year)**	**Primary end points**	**Secondary end points**	**Evaluated method for clinical remission**	**Evaluated method for endoscopic remission**	**Evaluated method for histological remission**	**Duration (weeks)**
Sandborn (2015)	clinical remission; endoscopic remission	AEs	Modified Mayo Disease	Mayo endoscopic subscore	—	6
Sandborn (2015*)	clinical remission; endoscopic remission	AEs	Modified Mayo Disease Activity Index score	Mayo endoscopic subscore	—	6
Crispino (2015)	clinical remission; endoscopic remission	AEs	Rachmilewitz Index	Baron *et al*. criteria	Truelove and Richards criteria	8
Kobayashi (2014)	endoscopic remission	AEs	—	Mayo endoscopic subscore	—	4
Watanabe (2013)	clinical remission; endoscopic remission	AEs	Sutherland Index	—	—	4
Hartmann (2010)	clinical remission; endoscopic remission	histopathological remission; AEs	Rachmilewitz Index	Löfberg Score	Floren	4
Andus (2010)	clinical remission; endoscopic remission	histopathological remission; AEs	Sutherland Index	Rachmilewitz Endoscopic Index	Riley *et al*.	6
Biancone (2007)	clinical remission	AEs	Rachmilewitz Index	—	—	8
Gionchetti (2005)	clinical remission	—	Schroeder Score or Mayo Score	Baron’s criteria	—	6
Hammond (2004)	clinical remission	AEs	Rachmilewitz Index	Rachmilewitz Endoscopic Index	Floren	4
Bar-Meir (2003)	clinical remission	AEs	Sutherland Index	Baron’s criteria	Floren	8
Malchow (2002)	clinical remission; endoscopic remission	AEs	Rachmilewitz Index	Rachmilewitz Endoscopic Index	—	4
Lindgren (2002)	clinical remission	AEs	—		Floren	8
Pokrotnieks(2000)	clinical remission; endoscopic remission	AEs	Rachmilewitz Index	Rachmilewitz Endoscopic Index	Floren	6
Franzè (1999)	clinical remission	—	—	—	—	4
Hanauer (1998)	clinical remission; endoscopic remission	AEs	Sutherland Index	Löfberg Score	Truelove and Richards criteria	6
Hanauer (1998*)	clinical remission; endoscopic remission	histopathological remission; AEs	PGA scores	The Siginoidoscopic Index	Truelove and Richards criteria	8
Campieri (1998)	clinical remission; endoscopic remission	AEs	—	Baron’s criteria	Truelove and Richards criteria	4
Mulder (1996)	endoscopical remission	AEs	Rachmilewitz Index	Löfberg Score	Truelove and Richards criteria	4
Lee (1996)	clinical remission; endoscopic remission	histopathological remission; AEs	Sutherland Index	Sutherland Index subscore	Riley *et al*.	4
Lemann (1995)	clinical remission; endoscopic remission	histopathological remission; AEs	Sutherland Index	Lémann Endoscopic Index	Floren	4
Lofberg (1994)	clinical remission; endoscopic remission	histopathological remission; AEs	—	Löfberg Score	Floren	8
Porro (1994)	clinical remission; endoscopic remission	histopathological remission; AEs	Truelove and Witts Severity Index	—	Floren	4
Farup (1994)	clinical remission	AEs	Rachmilewitz Index	—	Friedman *et al*.	4
Campieri (1991)	clinical remission; endoscopic remission	histopathological remission; AEs	Truelove & Richard	Truelove & Richard	Truelove & Richard	4
Campieri (1990)	clinical remission; endoscopic remission	histopathological remission; AEs	Truelove & Richard	Truelove & Richard	Truelove & Richard	4
Campieri (1990*)	clinical remission; endoscopic remission	histopathological remission; AEs	Truelove & Richard	Baron’s criteria	Truelove & Richard	4
Danielsson (1987)	endoscopical remission	AEs	—	Truelove & Richard	Floren	4
Williams (1987)	clinical remission	AEs	Sutherland Index	—	—	6
Sutherland (1987)	clinical remission	AEs	Sutherland Index	—	—	6
Sutherland(1987*)	clinical remission	AEs	Sutherland Index	—	—	6
Binder (1987)	clinical remission; endoscopic remission	AEs	Binder	Binder	—	4
Campieri (1981)	clinical remission; endoscopic remission	AEs	Truelove & Richard	Truelove & Richard	—	2
Jones (1971)	clinical remission	AEs	—	Baron’s criteria	—	4

5-ASA, 5-aminosalicylic acid; BDP, beclomethasone dipropionate; UC, Ulcerative colitis; UP, Ulcerative proctitis; UPS, Ulcerative proctosigmoiditis.

**Table 2:** Comparison of outcomes between traditional meta-analysis and Bayesian network meta-analysis.

Treatment Comparisons	Results of Pair-Wise Meta-Analysis	*I*^2^ (%)	Results of Network Meta-Analysis
**Clinical remission**			
Budesonide ≥4 mg/d **v** placebo	2.72 (1.86, 3.99)	24.6	2.88 (1.99, 4.26)
5-ASA 4 g/d **v** BDP 3 mg/d	0.74 (0.13, 4.02)	79.1	1.00 (0.58, 1.63)
5-ASA 4 g/d **v** 5-ASA 1.5 to 2.0 g/d + BDP 3 mg/d	0.21 (0.06, 0.84)	—	0.39 (0.05, 1.11)
BDP 3 mg/d **v** 5-ASA 1.5 to 2.0 g/d + BDP 3 mg/d	0.73 (0.15, 3.49)	—	0.40 (0.06, 1.26)
5-ASA 1 g/d **v** placebo	6.22 (3.86, 10.01)	0	5.57 (3.70, 8.23)
5-ASA 4 g/d **v** Budesonide 2 to 2.3 mg/d	1.95 (1.06, 3.60)	—	2.84 (1.78, 4.17)
5-ASA 1.5 to 2.0 g/d **v** 5-ASA 1 g/d	1.11 (0.77, 1.61)	0	1.15 (0.79, 1.61)
5-ASA 1.5 to 2.0 g/d **v** BDP 3 mg/d	1.27 (0.50, 3.22)	—	1.00 (0.58, 1.64)
5-ASA 1 g/d **v** BDP 3 mg/d	0.71 (0.38, 1.33)	—	0.88 (0.51, 1.39)
Budesonide 2 to 2.3 mg/d **v** Betamethasone 5 mg/d	0.16 (0.04, 0.73)	—	0.42 (0.16, 0.88)
Budesonide 2 to 2.3 mg/d **v** Hydrocortisone 100 mg/d	1.04 (0.63, 1.71)	—	1.42 (0.75, 2.59)
5-ASA 4 g/d **v** 5-ASA 1.5 to 2.0 g/d	1.06 (0.72, 1.58)	0	1.02 (0.68, 1.45)
Budesonide≥ 4 mg/d **v** Budesonide 2 to 2.3 mg/d	1.50 (0.92, 2.46)	0	1.30 (0.80, 1.98)
5-ASA 1.5 to 2.0 g/d **v** placebo	7.11 (3.48, 14.52)	53.8	6.30 (4.33, 9.08)
Budesonide 0.5 mg/d **v** placebo	2.00 (0.87, 4.63)	—	1.65 (0.69, 3.17)
Budesonide 2 to 2.3 mg/d **v** placebo	2.79 (1.22, 6.37)	—	2.30 (1.50, 3.47)
Budesonide 2 to 2.3 mg/d **v** Budesonide 0.5 mg/d	1.39 (0.65, 3.00)	—	1.64 (0.71, 3.29)
Budesonide ≥4 mg/d **v** Budesonide 0.5 mg/d	2.44 (1.16, 5.17)	—	2.06 (0.90, 4.12)
5-ASA 4 g/d **v** placebo	5.62 (3.28, 9.65)	23.9	6.35 (4.33, 9.26)
5-ASA 4 g/d **v** 5-ASA 1 g/d	1.03 (0.58, 1.81)	0	1.16 (0.77, 1.71)
BDP 3 mg/d **v** Prednisolone ≥30 mg/d	0.90 (0.45, 1.78)	—	1.16 (0.54, 2.24)
5-ASA 1.5 to 2.0 g/d **v** Prednisolone 20 to 25 mg/d	2.47 (1.53, 3.97)	—	2.30 (1.37, 3.62)
5-ASA 1 g/d **v** Budesonide 2 to 2.3 mg/d	2.43 (1.05, 5.61)	—	2.50 (1.58, 3.98)
Budesonide 2 to 2.3 mg/d **v** Prednisolone ≥30 mg/d	0.60 (0.22, 1.65)	—	0.41 (0.18, 0.84)
Budesonide 2 to 2.3 mg/d **v** Prednisolone 20 to 25 mg/d	1.10 (0.47, 2.61)	—	0.84 (0.46, 1.36)
5-ASA 1 g/d **v** Hydrocortisone 356 mg/d	1.36 (0.55, 3.40)	—	1.61 (0.48, 3.91)
5-ASA 1 g/d **v** Prednisolone 20 to 25 mg/d	1.68 (0.78, 3.62)	—	2.04 (1.22, 3.23)
5-ASA 4 g/d **v** Hydrocortisone 100 mg/d	10.25 (2.73, 38.45)	—	4.00 (1.99, 7.59)
Betamethasone 5 mg/d **v** Prednisolone 20 to 25 mg/d	1.47 (0.66, 3.31)	—	2.30 (0.96, 4.66)
**Endoscopic remission**			
Budesonide ≥4 mg/d **v** placebo	2.29 (1.42, 3.71)	51.6	2.55 (1.55, 4.12)
5-ASA 4 g/d **v** BDP 3 mg/d	1.22 (0.51, 2.94)	—	1.27 (0.60, 2.51)
5-ASA 4 g/d **v** 5-ASA 1.5 to 2.0 g/d + BDP 3 mg/d	0.60 (0.24, 1.46)	—	0.72 (0.44, 1.13)
BDP 3 mg/d **v** 5-ASA 1.5 to 2.0 g/d + BDP 3 mg/d	0.55 (0.26, 1.17)	0	0.63 (0.30, 1.17)
5-ASA 1 g/d **v** placebo	6.45 (4.23, 9.82)	0	4.97 (3.21, 7.51)
5-ASA 4 g/d **v** Budesonide 2 to 2.3 mg/d	1.19 (0.66, 2.16)	—	1.60 (0.89, 2.66)
5-ASA 1.5 to 2.0 g/d **v** 5-ASA 1 g/d	1.24 (0.86, 1.78)	0	1.01 (0.64, 1.48)
5-ASA 4 g/d **v** 5-ASA 1.5 to 2.0 g/d	1.08 (0.73, 1.61)	0	1.12 (0.68, 1.73)
5-ASA 1.5 to 2.0 g/d **v** placebo	4.49 (2.61, 7.73)	26.3	4.89 (3.22, 7.16)
Budesonide 0.5 mg/d **v** placebo	1.36 (0.52, 3.56)	—	1.23 (0.39, 2.98)
Budesonide 2 to 2.3 mg/d **v** placebo	3.15 (1.29, 7.70)	—	3.53 (1.90, 5.95)
Budesonide 2 to 2.3 mg/d **v** Budesonide 0.5 mg/d	2.32 (0.99, 5.46)	—	3.57 (1.20, 8.89)
Budesonide ≥4 mg/d **v** Budesonide 0.5 mg/d	3.42 (1.49, 7.86)	—	2.58 (0.91, 5.92)
Budesonide ≥4 mg/d **v** Budesonide 2 to 2.3 mg/d	1.47 (0.70, 3.11)	—	0.77 (0.37, 1.48)
5-ASA 4 g/d **v** placebo	6.86 (3.53, 13.34)	0	5.36 (3.26, 8.38)
5-ASA 4 g/d **v** 5-ASA 1 g/d	1.34 (0.76, 2.36)	0	1.11 (0.65, 1.76)
BDP 3 mg/d **v** Prednisolone ≥30 mg/d	1.21 (0.60, 2.46)	—	1.58 (0.68, 3.36)
5-ASA 1.5 to 2.0 g/d **v** 5-ASA 1.5 to 2.0 g/d + BDP 3 mg/d	0.18 (0.03, 1.02)	—	0.66 (0.44, 0.97)
5-ASA 1.5 to 2.0 g/d **v** BDP 3 mg/d	0.25 (0.04, 1.40)	—	1.17 (0.54, 2.16)
5-ASA 1.5 to 2.0 g/d **v** Prednisolone 20 to 25 mg/d	1.47 (0.91, 2.38)	—	1.40 (0.74, 2.34)
5-ASA 1 g/d **v** Budesonide 2 to 2.3 mg/d	0.95 (0.28, 3.20)	—	1.50 (0.81, 2.59)
Budesonide 2 to 2.3 mg/d **v** Prednisolone ≥30 mg/d	1.33 (0.22, 7.93)	83.1	1.20 (0.58, 2.22)
Budesonide 2 to 2.3 mg/d **v** Prednisolone 20 to 25 mg/d	0.71 (0.22, 2.25)	—	1.01 (0.49, 1.86)
5-ASA 1 g/d **v** Prednisolone 20 to 25 mg/d	1.36 (0.65, 2.86)	—	1.43 (0.74, 2.42)
5-ASA 4 g/d **v** Hydrocortisone 100 mg/d	11.29 (3.02, 42.28)	—	3.49 (0.91, 9.87)
**Histopathological remission**			
5-ASA 4 g/d **v** Budesonide 2 to 2.3 mg/d	1.25 (0.72, 2.17)	—	1.67 (0.79, 3.41)
5-ASA 1.5 to 2.0 g/d **v** 5-ASA 1 g/d	1.45 (1.01, 2.07)	0	1.42 (0.78, 2.26)
5-ASA 4 g/d **v** 5-ASA 1 g/d	1.51 (0.86, 2.64)	0	1.44 (0.72, 2.66)
5-ASA 1 g/d **v** placebo	4.36 (1.82, 10.42)	14.9	6.33 (2.67, 13.32)
5-ASA 4 g/d **v** 5-ASA 1.5 to 2.0 g/d	1.15 (0.66, 1.99)	0	1.05 (0.52, 1.90)
5-ASA 1.5 to 2.0 g/d **v** placebo	5.75 (2.11, 15.70)	31	8.56 (3.79, 17.29)
5-ASA 4 g/d **v** placebo	9.49 (2.14, 42.04)	32.7	8.64 (3.68, 20.26)
5-ASA 1.5 to 2.0 g/d **v** Prednisolone 20 to 25 mg/d	1.36 (0.80, 2.33)	—	1.50 (0.62, 3.18)
5-ASA 1 g/d **v** Budesonide 2 to 2.3 mg/d	1.80 (0.55, 5.90)	—	1.26 (0.50, 2.84)
Budesonide 2 to 2.3 mg/d **v** Prednisolone ≥30 mg/d	0.57 (0.16, 2.04)	—	0.75 (0.11, 2.40)
Budesonide 2 to 2.3 mg/d **v** Prednisolone 20 to 25 mg/d	0.81 (0.23, 2.89)	—	1.00 (0.32, 2.25)
**Adverse events**			
Budesonide ≥4 mg/d **v** placebo	1.50 (1.10, 2.05)	0	1.56 (0.84, 2.69)
5-ASA 1 g/d **v** placebo	0.96 (0.39, 2.37)	0	1.31 (0.59, 2.57)
5-ASA 4 g/d **v** Budesonide 2 to 2.3 mg/d	0.80 (0.46, 1.41)	—	0.73 (0.35, 1.32)
5-ASA 1.5 to 2.0 g/d **v** 5-ASA 1 g/d	1.15 (0.70, 1.87)	—	1.05 (0.54, 1.66)
5-ASA 1.5 to 2.0 g/d **v** BDP 3 mg/d	0.68 (0.31, 1.48)	0	0.91 (0.41, 1.75)
5-ASA 1 g/d **v** BDP 3 mg/d	1.19 (0.52, 2.75)	—	0.91 (0.38, 1.82)
Budesonide 2 to 2.3 mg/d **v** Betamethasone 5 mg/d	0.60 (0.16, 2.28)	—	1.02 (0.29, 2.75)
Budesonide 2 to 2.3 mg/d **v** Hydrocortisone 100 mg/d	0.67 (0.39, 1.13)	—	0.64 (0.24, 1.25)
5-ASA 4 g/d **v** 5-ASA 1.5 to 2.0 g/d	0.24 (0.08, 0.76)	—	0.74 (0.30, 1.43)
Budesonide ≥4 mg/d **v** Budesonide 2 to 2.3 mg/d	1.24 (0.75, 2.07)	0	1.31 (0.69, 2.29)
5-ASA 1.5 to 2.0 g/d **v** placebo	0.52 (0.18, 1.53)	—	1.30 (0.59, 2.34)
Budesonide 0.5 mg/d **v** placebo	1.36 (0.63, 2.94)	—	1.46 (0.59, 3.31)
Budesonide 2 to 2.3 mg/d **v** placebo	1.30 (0.60, 2.82)	—	1.25 (0.63, 2.08)
Budesonide 2 to 2.3 mg/d **v** Budesonide 0.5 mg/d	0.95 (0.44, 2.05)	—	1.01 (0.36, 2.14)
Budesonide ≥4 mg/d **v** Budesonide 0.5 mg/d	1.14 (0.54, 2.41)	—	1.25 (0.45, 2.69)
BDP 3 mg/d **v** Prednisolone ≥30 mg/d	1.28 (0.42, 3.87)	—	1.35 (0.40, 3.84)
BDP 3 mg/d **v** 5-ASA 1.5 to 2.0 g/d + BDP 3 mg/d	0.72 (0.18, 2.93)	—	0.83 (0.15, 2.80)
5-ASA 1.5 to 2.0 g/d **v** 5-ASA 1.5 to 2.0 g/d + BDP 3 mg/d	0.51 (0.12, 2.19)	—	0.72 (0.13, 2.23)
5-ASA 1.5 to 2.0 g/d **v** Prednisolone 20 to 25 mg/d	1.49 (0.93, 2.40)	—	1.72 (0.77, 3.33)
5-ASA 1 g/d **v** Budesonide 2 to 2.3 mg/d	0.31 (0.03, 3.12)	—	1.12 (0.46, 2.42)
Budesonide 2 to 2.3 mg/d **v** Prednisolone ≥30 mg/d	0.85 (0.07, 10.07)	38.4	1.15 (0.27, 3.29)
Budesonide 2 to 2.3 mg/d **v** Prednisolone 20 to 25 mg/d	—	—	1.76 (0.61, 4.02)
5-ASA 1 g/d **v** Hydrocortisone 356 mg/d	0.91 (0.27, 3.13)	—	1.23 (0.23, 4.11)
5-ASA 4 g/d **v** placebo	0.81 (0.31, 2.07)	—	0.88 (0.42, 1.53)
5-ASA 1 g/d **v** Prednisolone 20 to 25 mg/d	2.53 (0.89, 7.16)	—	1.73 (0.75, 3.49)
5-ASA 4 g/d **v** Hydrocortisone 100 mg/d	0.18 (0.01, 3.91)	—	0.47 (0.13, 1.07)
Betamethasone 5 mg/d **v** Prednisolone 20 to 25 mg/d	1.27 (0.40, 4.08)	—	2.13 (0.61, 5.80)

5-ASA, 5-aminosalicylic acid; BDP, beclomethasone dipropionate.

**Table 3:** Treatment efficacy and safety estimates from Bayesian network meta-analyses.

**Clinical remission**													
5-ASA 4 g/d													
1.02 (0.68, 1.45)	5-ASA 1.5 to 2.0 g/d												
1.16 (0.77, 1.71)	1.15 (0.79, 1.61)	5-ASA 1 g/d											
**2.27 (1.35, 3.54)**	**2.26 (1.35, 3.62)**	**2.00 (1.15, 3.20)**	Budesonide ≥ 4 mg/d										
**2.84 (1.78, 4.17)**	**2.83 (1.76, 4.40)**	**2.50 (1.58, 3.98)**	1.30 (0.80, 1.98)	Budesonide 2 to 2.3 mg/d									
**4.59 (1.90, 9.75)**	**4.57 (1.85, 9.60)**	**4.03 (1.69, 8.66)**	2.06 (0.90, 4.12)	1.64 (0.71, 3.29)	Budesonide 0.5 mg/d								
1.00 (0.58, 1.63)	1.00 (0.58, 1.64)	0.88 (0.51, 1.39)	**0.46 (0.24, 0.81)**	**0.36 (0.20, 0.59)**	**0.26 (0.09, 0.57)**	BDP 3 mg/d							
1.18 (0.44, 2.54)	1.17 (0.43, 2.47)	1.03 (0.38, 2.21)	0.54 (0.20, 1.22)	**0.42 (0.16, 0.88)**	**0.30 (0.08, 0.77)**	1.24 (0.42, 2.89)	Betamethasone 5 mg/d						
1.87 (0.52, 4.68)	1.85 (0.51, 4.45)	1.61 (0.48, 3.91)	0.87 (0.23, 2.24)	0.68 (0.18, 1.76)	0.48 (0.10, 1.42)	1.95 (0.52, 5.27)	1.91 (0.39, 5.79)	Hydrocortisone 356 mg/d					
**4.00 (1.99, 7.59)**	**4.01 (1.89, 7.83)**	**3.54 (1.66, 7.13)**	1.84 (0.85, 3.85)	1.42 (0.75, 2.59)	1.01 (0.35, 2.41)	**4.21 (1.86, 8.61)**	**4.09 (1.28, 10.35)**	2.94 (0.68, 8.61)	Hydrocortisone 100 mg/d				
1.15 (0.49, 2.36)	1.14 (0.48, 2.36)	1.00 (0.42, 2.01)	0.53 (0.21, 1.13)	**0.41 (0.18, 0.84)**	**0.29 (0.08, 0.73)**	1.16 (0.54, 2.24)	1.17 (0.33, 3.12)	0.82 (0.18, 2.35)	**0.32 (0.10, 0.70)**	Prednisolone ≥30 mg/d			
**2.34 (1.28, 3.92)**	**2.30 (1.37, 3.62)**	**2.04 (1.22, 3.23)**	1.07 (0.55, 1.87)	0.84 (0.46, 1.36)	0.59 (0.22, 1.25)	**2.45 (1.25, 4.44)**	2.30 (0.96, 4.66)	1.68 (0.47, 4.43)	0.64 (0.27, 1.20)	2.33 (0.89, 5.05)	Prednisolone 20 to 25 mg/d		
0.39 (0.05, 1.11)	0.39 (0.05, 1.16)	0.35 (0.05, 1.08)	**0.18 (0.02, 0.57)**	**0.14 (0.02, 0.44)**	**0.10 (0.01, 0.34)**	0.40 (0.06, 1.26)	0.40 (0.04, 1.45)	0.29 (0.03, 1.10)	**0.11 (0.01, 0.37)**	0.39 (0.05, 1.24)	**0.18 (0.02, 0.57)**	5-ASA 1.5 to 2.0 g/d + BDP 3 mg/d	
**6.35 (4.33, 9.26)**	**6.30 (4.33, 9.08)**	**5.57 (3.70, 8.23)**	**2.88 (1.99, 4.26)**	**2.30 (1.50, 3.47)**	1.60 (0.69, 3.17)	**6.69 (3.78, 11.54)**	**6.52 (2.47, 14.58)**	**4.60 (1.31, 12.06)**	1.75 (0.80, 3.20)	**6.38 (2.65, 13.08)**	**2.88 (1.68, 4.84)**	**29.22 (5.15, 117.49)**	Placebo
**Endoscopic remission**													
5-ASA 4 g/d													
1.12 (0.68, 1.73)	5-ASA 1.5 to 2.0 g/d												
1.11 (0.65, 1.76)	1.01 (0.64, 1.48)	5-ASA 1 g/d											
**2.22 (1.10, 4.11)**	**2.04 (1.01, 3.46)**	**2.07 (1.00, 3.74)**	Budesonide ≥4 mg/d										
1.60 (0.89, 2.66)	1.48 (0.76, 2.53)	1.50 (0.81, 2.59)	0.77 (0.37, 1.48)	Budesonide 2 to 2.3 mg/d									
**5.58 (1.66, 14.37)**	**5.13 (1.49, 12.76)**	**5.19 (1.55, 12.82)**	2.58 (0.91, 5.92)	**3.57 (1.20, 8.89)**	Budesonide 0.5 mg/d								
1.27 (0.60, 2.51)	1.17 (0.54, 2.16)	1.19 (0.54, 2.33)	0.61 (0.26, 1.27)	0.83 (0.37, 1.64)	**0.29 (0.08, 0.77)**	BDP 3 mg/d							
3.49 (0.91, 9.87)	3.33 (0.75, 10.20)	3.33 (0.76, 9.54)	1.77 (0.35, 6.08)	2.39 (0.54, 7.42)	0.85 (0.13, 3.18)	3.18 (0.60, 10.50)	Hydrocortisone 100 mg/d						
1.89 (0.76, 3.94)	1.73 (0.71, 3.41)	1.76 (0.71, 3.53)	0.92 (0.35, 2.10)	1.20 (0.58, 2.22)	0.43 (0.10, 1.06)	1.58 (0.68, 3.36)	0.77 (0.12, 2.58)	Prednisolone ≥30 mg/d					
1.55 (0.79, 2.84)	1.40 (0.74, 2.34)	1.43 (0.74, 2.42)	0.75 (0.32, 1.51)	1.01 (0.49, 1.86)	**0.36 (0.09, 1.00)**	1.36 (0.51, 2.98)	0.63 (0.12, 1.94)	0.94 (0.34, 2.16)	Prednisolone 20 to 25 mg/d				
0.72 (0.44, 1.13)	**0.66 (0.44, 0.97)**	0.67 (0.43, 1.02)	**0.35 (0.21, 0.56)**	**0.48 (0.26, 0.81)**	**0.17 (0.05, 0.40)**	0.63 (0.30, 1.17)	**0.29 (0.06, 0.84)**	**0.44 (0.18, 0.91)**	**0.51 (0.26, 0.91)**	5-ASA 1.5 to 2.0 g/d + BDP 3 mg/d			
**5.36 (3.26, 8.38)**	**4.89 (3.22, 7.16)**	**4.97 (3.21, 7.51)**	**2.55 (1.55, 4.12)**	**3.53 (1.90, 5.95)**	1.23 (0.39, 2.98)	**4.66 (2.21, 8.67)**	2.18 (0.47, 6.21)	**3.25 (1.35, 6.73)**	**3.76 (1.92, 6.76)**	**17.00 （5.21, 41.18)**	Placebo		
**Histopathological remission**													
5-ASA 4 g/d													
1.05 (0.52, 1.90)	5-ASA 1.5 to 2.0 g/d												
1.44 (0.72, 2.66)	1.42 (0.78, 2.26)	5-ASA 1 g/d											
1.67 (0.79, 3.41)	1.73 (0.73, 3.82)	1.26 (0.50, 2.84)	Budesonide 2 to 2.3 mg/d										
1.25 (0.14, 4.38)	1.37 (0.14, 4.63)	0.96 (0.10, 3.33)	0.75 (0.11, 2.40)	Prednisolone ≥30 mg/d									
1.55 (0.56, 3.90)	1.50 (0.62, 3.18)	1.12 (0.40, 2.79)	1.00 (0.32, 2.25)	2.77 (0.29, 10.19)	Prednisolone 20 to 25 mg/d								
**8.64 (3.68, 20.26)**	**8.56 (3.79, 17.29)**	**6.33 (2.67, 13.32)**	**5.75 (1.87, 13.60)**	**14.68 (1.71, 62.94)**	**6.82 (1.93, 18.55)**	Placebo							
**Adverse events**													
5-ASA 4 g/d													
0.74 (0.30, 1.43)	5-ASA 1.5 to 2.0 g/d												
0.75 (0.27, 1.55)	1.05 (0.54, 1.66)	5-ASA 1 g/d											
0.60 (0.24, 1.15)	0.90 (0.34, 1.85)	0.91 (0.33, 2.13)	Budesonide ≥4 mg/d										
0.73 (0.35, 1.32)	1.11 (0.45, 2.19)	1.12 (0.46, 2.42)	1.31 (0.69, 2.29)	Budesonide 2 to 2.3 mg/d									
0.71 (0.22, 1.62)	1.08 (0.28, 2.71)	1.09 (0.30, 2.73)	1.25 (0.45, 2.69)	1.01 (0.36, 2.14)	Budesonide 0.5 mg/d								
0.65 (0.20, 1.64)	0.91 (0.41, 1.75)	0.91 (0.38, 1.82)	1.21 (0.36, 3.22)	0.94 (0.31, 2.27)	1.13 (0.26, 3.21)	BDP 3 mg/d							
0.71 (0.17, 1.82)	1.05 (0.29, 2.74)	1.06 (0.27, 2.82)	1.31 (0.33, 3.54)	1.02 (0.29, 2.75)	1.24 (0.26, 3.85)	1.31 (0.29, 3.90)	Betamethasone 5 mg/d						
0.91 (0.11, 3.12)	1.29 (0.20, 4.41)	1.23 (0.23, 4.11)	1.67 (0.19, 6.05)	1.32 (0.18, 4.66)	1.51 (0.16, 5.50)	1.54 (0.23, 5.58)	1.68 (0.16, 6.31)	Hydrocortisone 356 mg/d					
0.47 (0.13, 1.07)	0.71 (0.18, 1.70)	0.71 (0.20, 1.90)	0.85 (0.23, 2.04)	0.64 (0.24, 1.25)	0.80 (0.18, 2.18)	0.88 (0.18, 2.35)	0.87 (0.16, 2.63)	0.98 (0.11, 3.46)	Hydrocortisone 100 mg/d				
0.82 (0.17, 2.56)	1.17 (0.31, 3.40)	1.18 (0.27, 3.44)	1.49 (0.28, 4.71)	1.15 (0.27, 3.29)	1.38 (0.24, 4.61)	1.35 (0.40, 3.84)	1.50 (0.24, 4.98)	1.63 (0.16, 6.52)	2.17 (0.36, 6.96)	Prednisolone ≥30 mg/d			
1.23 (0.39, 2.88)	1.72 (0.77, 3.33)	1.73 (0.75, 3.49)	2.25 (0.74, 5.22)	1.76 (0.61, 4.02)	2.11 (0.55, 6.25)	2.14 (0.76, 5.19)	2.13 (0.61, 5.80)	2.46 (0.34, 8.36)	3.30 (0.82, 9.67)	2.08 (0.46, 6.10)	Prednisolone 20 to 25 mg/d		
0.52 (0.06, 1.77)	0.72 (0.13, 2.23)	0.73 (0.12, 2.30)	0.97 (0.12, 3.54)	0.76 (0.11, 2.69)	0.91 (0.11, 3.55)	0.83 (0.15, 2.80)	1.03 (0.09, 4.22)	1.01 (0.06, 4.57)	1.51 (0.15, 5.77)	0.83 (0.10, 3.66)	0.48 (0.06, 1.69)	5-ASA 1.5 to 2.0 g/d + BDP 3 mg/d	
0.88 (0.42, 1.53)	1.30 (0.59, 2.34)	1.31 (0.59, 2.57)	1.56 (0.84, 2.69)	1.25 (0.63, 2.08)	1.46 (0.59, 3.31)	1.62 (0.56, 3.76)	1.63 (0.46, 4.25）	1.90 (0.28, 7.09)	2.34 (0.81, 5.77)	1.54 (0.36, 4.34)	0.84 (0.30, 1.81)	3.15 (0.47, 11.43)	Placebo

The efficacy was estimated in the triangle, comparing column-defining with row-defining treatments. The estimates of effects were summarized as odds ratios (ORs) with their corresponding 95% credible intervals (CrIs) respectively. For the efficacy assessment, ORs greater than 1 favor the column-defining treatment, while for adverse effects, ORs greater than 1 favor the row-defining treatment. Results with significant statistical differences are shown in bold.5-ASA, 5-aminosalicylic acid; BDP, beclomethasone dipropionate.

**Table 4:** Evaluation of model fit in the included studies.

Outcome	Residual deviance	Number of data points
Clinical remission	75.47	70
Endoscopic remission	57.91	55
Histopathological remission	26.42	25
Adverse events	54.31	55

The model was considered to provide an adequate fit to the data if the mean of the residual deviance approximated the number of data points.
