# Supplementary material for: Efficacy and safety of rectal 5-aminosalicylic acid versus corticosteroids in active distal ulcerative colitis: a systematic review and network meta-analysis
Source: Sci Rep. 2017 Apr 25;7:46693. doi: 10.1038/srep46693 (PMC5404224; doi:10.1038/srep46693)
Supplement: Supplementary Information [file srep46693-s1.pdf]

Efficacy and safety of rectal 5-aminosalicylic acid versus corticosteroids in active distal ulcerative colitis: a systematic review and network meta-analysis

Xiaojing Zhao[1,*], Changcheng Zhou[2,*], Jingjing Ma[1,*], Yunjuan Zhu[1], Min Sun[3], Peixue Wang[1], Yi Zhang[1], Haiqin Ma[1], Hongjie Zhang[1]

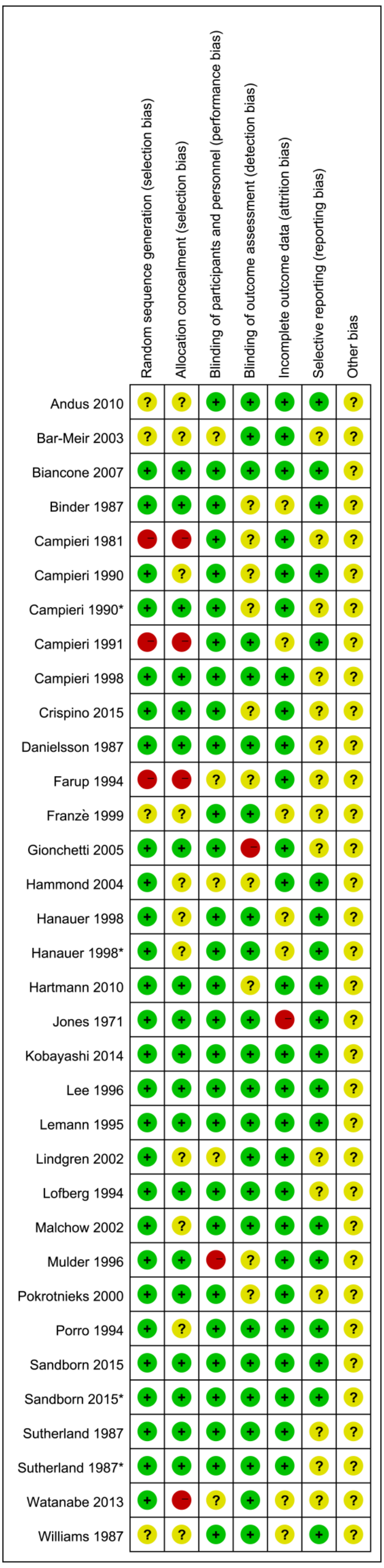

Figure S1. Risk of bias summary. Review judgments about the risks of bias items for all included studies. (+) = low risk, (?) = unclear, (-) = high risk.

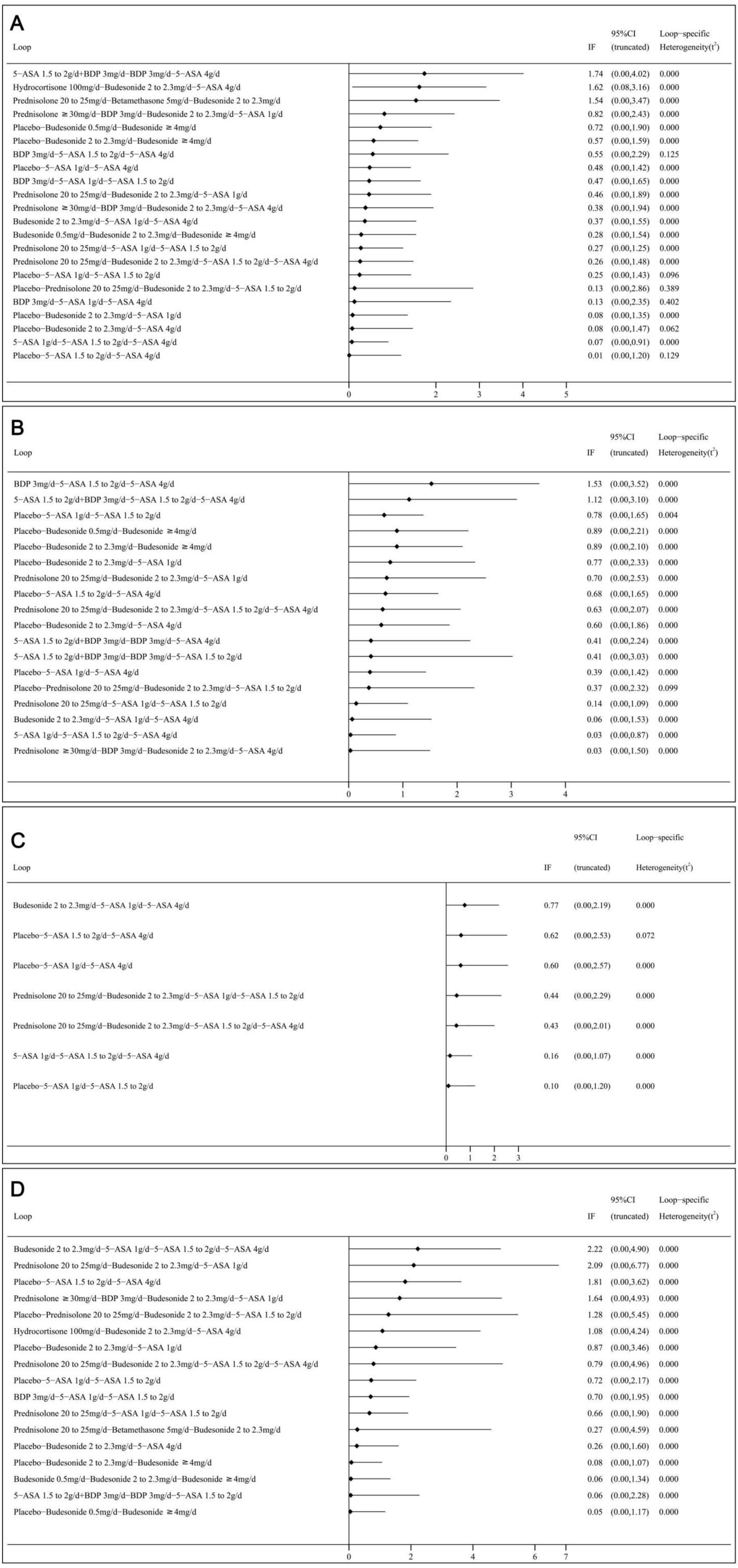

Figure S2. Evaluation of consistency. (A) Clinical remission. (B) Endoscopic remission. (C) Histopathological remission. (D) Adverse events. 5-ASA, 5-aminosalicylic acid; BDP, beclomethasone dipropionate.

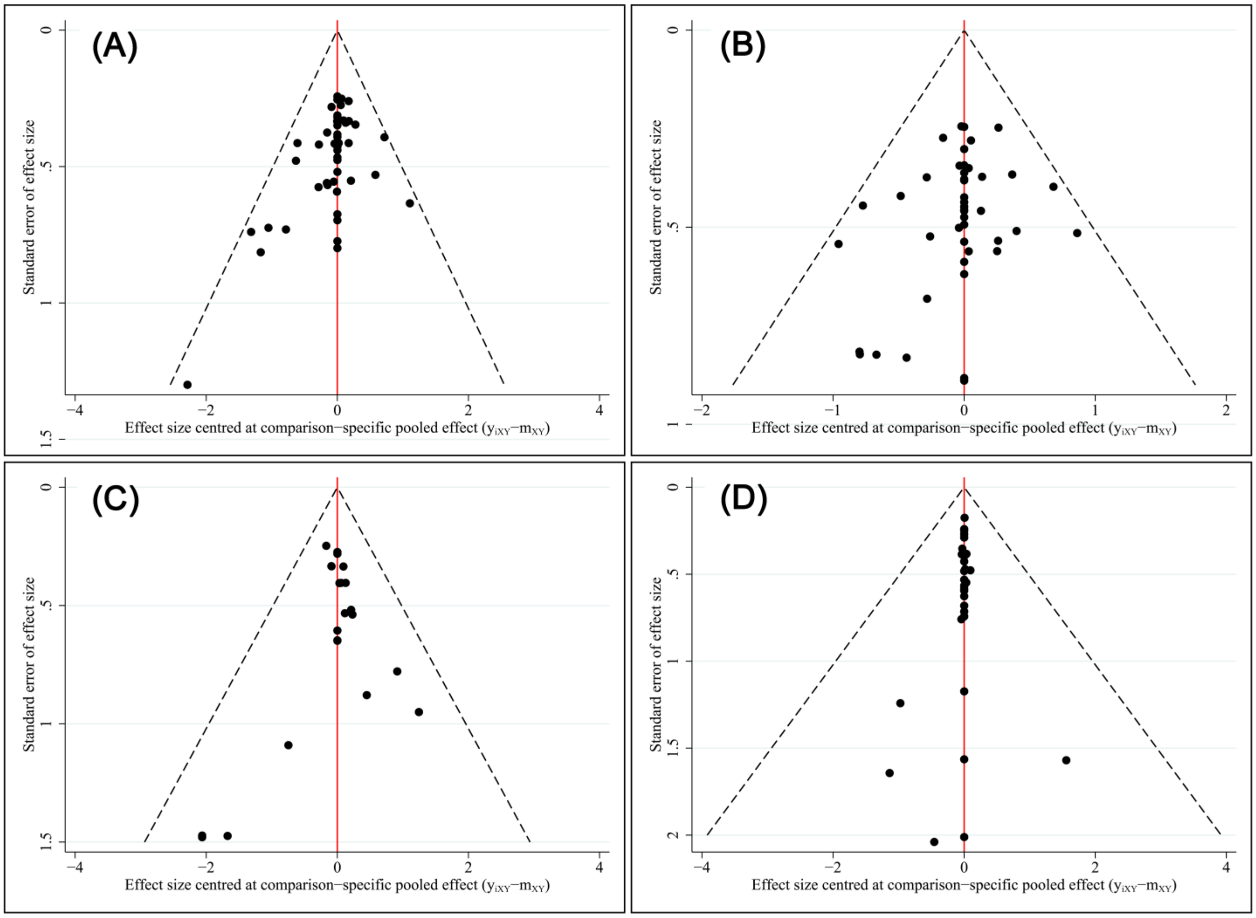

Figure S3. Comparison-adjusted funnel plot for publication bias. (A) clinical remission, (B) endoscopic remission, (C) histopathological remission and (D) incidence of adverse events.

Comparison	Direct evidence		Indirect evidence		Network meta-analysis	
	OR(95%CI)	Quality of evidence	OR(95%CrI)	Quality of evidence	OR(95%CrI)	Quality of evidence
Budesonide ⩾4mg/d **v** placebo	2.72(1.86, 3.99)	Moderate‡	3.13(0.90, 9.86)	Very low**‡‡	2.88(1.99, 4.26)	Moderate §§
5-ASA 4g/d v BDP 3mg/d	0.74(0.13, 4.02)	Very low*†‡	0.91(0.42, 2.02)	Low**	1.00(0.58, 1.63)	Low
5-ASA 4g/d v 5-ASA 1.5 to 2.0g/d +BDP 3mg/d	0.21(0.06, 0.84)	Low†‡	0.64(0.11, 3.55)	Very low**‡‡	0.39(0.05, 1.11)	Low
BDP 3mg/d v 5-ASA 1.5 to 2.0g/d +BDP 3mg/d	0.73(0.15, 3.49)	Low†‡	0.24(0.05, 1.13)	Low**	0.40(0.06, 1.26)	Low
5-ASA 1g/d v placebo	6.22(3.86, 10.01)	Very low*§‡	4.98(2.59, 10.56)	Low**	5.57(3.70, 8.23)	Low §§
5-ASA 4g/d v Budesonide 2 to 2.3mg/d	1.95(1.06, 3.60)	Low*‡	2.80(1.58, 5.01)	Very low**‡‡	2.84(1.78, 4.17)	Low §§
5-ASA 1.5 to 2.0g/d v 5-ASA 1g/d	1.11(0.77, 1.61)	Moderate*	1.37(0.67, 3.15)	Moderate ¶	1.15(0.79, 1.61)	Moderate
5-ASA 1.5 to 2.0g/d v BDP 3mg/d	1.27(0.50, 3.22)	Moderate‡	1.06(0.53, 2.14)	Very low**	1.00(0.58, 1.64)	Moderate
5-ASA 1g/d v BDP 3mg/d	0.71(0.38, 1.33)	Moderate‡	1.04(0.45, 2.17)	Moderate ¶	0.88(0.51, 1.39)	Moderate
Budesonide 2 to 2.3mg/d **v** Betamethasone 5mg/d	0.16(0.04, 0.73)	Low*‡	0.54(0.16, 1.90)	Low**	0.42(0.16, 0.88)	Low §§
Budesonide 2 to 2.3mg/d **v** Hydrocortisone 100mg/d	1.04(0.63, 1.71)	Moderate*	5.50(1.18, 31.16)	Low**	1.42(0.75, 2.59)	Moderate
5-ASA 4g/d v 5-ASA 1.5 to 2.0g/d	1.06(0.72, 1.58)	Low*†	0.79(0.40, 1.53)	Very low**	1.02(0.68, 1.45)	Low
Budesonide ⩾4mg/d **v** Budesonide 2 to 2.3mg/d	1.50(0.92, 2.46)	Low*‡	1.05(0.41, 2.52)	Low**	1.30(0.80, 1.98)	Low
5-ASA 1.5 to 2.0g/d v placebo	7.11(3.48, 14.52)	Low*†	7.70(3.74, 16.20)	Very low**‡‡	6.30(4.33, 9.08)	Low §§
Budesonide 0.5mg/d **v** placebo	2.00(0.87, 4.63)	Low*‡	1.31(0.56, 3.03)	Low**	1.65(0.69, 3.17)	Low
Budesonide 2 to 2.3mg/d **v** placebo	2.79(1.22, 6.37)	Low*‡	2.17(1.21, 3.87)	Low**	2.30(1.50, 3.47)	Low §§
Budesonide 2 to 2.3mg/d **v** Budesonide 0.5mg/d	1.39(0.65, 3.00)	Low*‡	1.45(0.59, 3.58)	Low**	1.64(0.71, 3.29)	Low
Budesonide ⩾4mg/d **v** Budesonide 0.5mg/d	2.44(1.16, 5.17)	Low*‡	1.61(0.67, 3.86)	Very low**	2.06(0.90, 4.12)	Low
5-ASA 4g/d v placebo	5.62(3.28, 9.65)	Low* §	5.15(2.78, 10.15)	Very low**‡‡	6.35(4.33, 9.26)	Low §§
5-ASA 4g/d v 5-ASA 1g/d	1.03(0.58, 1.81)	Very low*§‡	0.91(0.52, 1.71)	Very low**	1.16(0.77, 1.71)	Very low
BDP 3mg/d **v** Prednisolone ⩾30mg/d	0.90(0.45, 1.78)	Moderate‡	1.92(0.43, 7.94)	Low**	1.16(0.54, 2.24)	Moderate
5-ASA 1.5 to 2.0g/d v Prednisolone 20 to 25mg/d	2.47(1.53, 3.97)	Moderate‡	2.19(0.97, 5.03)	Moderate ¶	2.30(1.37, 3.62)	Moderate §§
5-ASA 1g/d v Budesonide 2 to 2.3mg/d	2.43(1.05, 5.61)	Moderate‡	2.63(1.45, 4.98)	Very low**‡‡	2.50(1.58, 3.98)	Moderate §§
Budesonide 2 to 2.3mg/d **v** Prednisolone ⩾30mg/d	0.60(0.22, 1.65)	Moderate‡	0.27(0.08, 0.91)	Very low**	0.41(0.18, 0.84)	Moderate §§
Budesonide 2 to 2.3mg/d **v** Prednisolone 20 to 25mg/d	1.10(0.47, 2.61)	Low*‡	0.60(0.28, 1.28)	Low**	0.84(0.46, 1.36)	Low
5-ASA 1g/d v Hydrocortisone 356mg/d	1.36(0.55, 3.40)	Low*‡	Not estimable	Not estimable††	1.61(0.48, 3.91)	Low
5-ASA 1g/d v Prednisolone 20 to 25mg/d	1.68(0.78, 3.62)	Moderate‡	2.02(0.95, 4.38)	Moderate ¶	2.04(1.22, 3.23)	Moderate §§
5-ASA 4g/d v Hydrocortisone 100mg/d	10.25(2.73, 38.45)	Low*‡	2.28(0.94, 5.45)	Low**	4.00(1.99, 7.59)	Low §§
Betamethasone 5mg/d **v** Prednisolone 20 to 25mg/d	1.47(0.66, 3.31)	Moderate‡	6.38(0.98, 42.27)	Low**	2.30(0.96, 4.66)	Moderate
5-ASA 1.5 to 2.0g/d+BDP 3mg/d v placebo	—	—	29.22(5.15, 117.49)	Very low**‡‡	29.22(5.15, 117.49)	Very low §§
Prednisolone 20 to 25mg/d v placebo	—	—	2.88(1.68, 4.84)	Low**	2.88(1.68, 4.84)	Low §§
Prednisolone 20 to 25mg/d v 5-ASA 1.5 to 2.0g/d+BDP 3mg/d	—	—	0.18(0.02, 0.57)	Low**	0.18(0.02, 0.57)	Low §§
Prednisolone ⩾30mg/d v placebo	—	—	6.38(2.65, 13.08)	Very low**‡‡	6.38(2.65, 13.08)	Very low §§
Prednisolone ≥30mg/d v 5-ASA 1.5 to 2.0g/d+BDP 3mg/d	—	—	0.39(0.05,1.24)	Very low**‡‡	0.39(0.05,1.24)	Very low
Prednisolone ⩾30mg/d v Prednisolone 20 to 25mg/d	—	—	2.33(0.89, 5.05)	Very low**‡‡	2.33(0.89, 5.05)	Very low
Hydrocortisone 100mg/d v placebo	—	—	1.75(0.80, 3.20)	Low**	1.75(0.80, 3.20)	Low
Hydrocortisone 100mg/d v 5-ASA 1.5 to 2.0g/d+BDP 3mg/d	—	—	0.11(0.01, 0.37)	Low**	0.11(0.01, 0.37)	Low §§
Hydrocortisone 100mg/d v Prednisolone 20 to 25mg/d	—	—	0.64(0.27, 1.20)	Very low**‡‡	0.64(0.27, 1.20)	Very low
Hydrocortisone 100mg/d v Prednisolone ⩾30mg/d	—	—	0.32(0.10, 0.70)	Low**	0.32(0.10, 0.70)	Low §§
Hydrocortisone 356mg/d v placebo	—	—	4.60(1.31, 12.06)	Low**	4.60(1.31, 12.06)	Low §§
Hydrocortisone 356mg/d v 5-ASA 1.5 to 2.0g/d+BDP 3mg/d	—	—	0.29(0.03, 1.10)	Low**	0.29(0.03, 1.10)	Low
Hydrocortisone 356mg/d v Prednisolone 20 to 25mg/d	—	—	1.68(0.47, 4.43)	Very low**‡‡	1.68(0.47, 4.43)	Very low
Hydrocortisone 356mg/d v Prednisolone ⩾30mg/d	—	—	0.82(0.18, 2.35)	Low**	0.82(0.18, 2.35)	Low
Hydrocortisone 356mg/d v Hydrocortisone 100mg/d	—	—	2.94(0.68, 8.61)	Low**	2.94(0.68, 8.61)	Low
Betamethasone 5mg/d v placebo	—	—	6.52(2.47, 14.58)	Low**	6.52(2.47, 14.58)	Low §§
Betamethasone 5mg/d v 5-ASA 1.5 to 2.0g/d+BDP 3mg/d	—	—	0.40(0.04, 1.45)	Very low**‡‡	0.40(0.04, 1.45)	Very low
Betamethasone 5mg/d v Prednisolone ⩾30mg/d	—	—	1.17(0.33, 3.12)	Low**	1.17(0.33, 3.12)	Low
Betamethasone 5mg/d v Hydrocortisone 100mg/d	—	—	4.09(1.28, 10.35)	Low**	4.09(1.28, 10.35)	Low §§
Betamethasone 5mg/d v Hydrocortisone 356mg/d	—	—	1.91(0.39, 5.79)	Low**	1.91(0.39, 5.79)	Low
BDP 3mg/d v placebo	—	—	6.69(3.78, 11.54)	Very low**‡‡	6.69(3.78, 11.54)	Very low §§
BDP 3mg/d v Prednisolone 20 to 25mg/d	—	—	2.45(1.25, 4.44)	Very low**‡‡	2.45(1.25, 4.44)	Very low §§

BDP 3mg/d v Hydrocortisone 100mg/d	—	—	4.21(1.86, 8.61)	Low**	4.21(1.86, 8.61)	Low §§
BDP 3mg/d v Hydrocortisone 356mg/d	—	—	1.95(0.52, 5.27)	Very low**‡‡	1.95(0.52, 5.27)	Very low
BDP 3mg/d v Betamethasone 5mg/d	—	—	1.24(0.42, 2.89)	Low**	1.24(0.42, 2.89)	Low
Budesonide 0.5mg/d v 5-ASA 1.5 to 2.0g/d+BDP 3mg/d	—	—	0.10(0.01, 0.38)	Very low**‡‡	0.10(0.01, 0.38)	Very low §§
Budesonide 0.5mg/d v Prednisolone 20 to 25mg/d	—	—	0.59(0.22, 1.25)	Very low**‡‡	0.59(0.22, 1.25)	Very low
Budesonide 0.5mg/d v Prednisolone ⩾30mg/d	—	—	0.29(0.08, 0.73)	Low**	0.29(0.08, 0.73)	Low §§
Budesonide 0.5mg/d v Hydrocortisone 100mg/d	—	—	1.01(0.35, 2.41)	Low**	1.01(0.35, 2.41)	Low
Budesonide 0.5mg/d v Hydrocortisone 356mg/d	—	—	0.48(0.10, 1.42)	Very low**‡‡	0.48(0.10, 1.42)	Very low
Budesonide 0.5mg/d v Betamethasone 5mg/d	—	—	0.30(0.08, 0.77)	Low**	0.30(0.08, 0.77)	Low §§
Budesonide 0.5mg/d v BDP 3mg/d	—	—	0.26(0.09, 0.57)	Low**	0.26(0.09, 0.57)	Low §§
Budesonide 2 to 2.3mg/d v 5-ASA 1.5 to 2.0g/d+BDP 3mg/d	—	—	0.14(0.02, 0.44)	Low**	0.14(0.02, 0.44)	Low §§
Budesonide 2 to 2.3mg/d v Hydrocortisone 356mg/d	—	—	0.68(0.18, 1.76)	Very low**‡‡	0.68(0.18, 1.76)	Very low
Budesonide 2 to 2.3mg/d v BDP 3mg/d	—	—	0.36(0.20, 0.59)	Low**	0.36(0.20, 0.59)	Low §§
Budesonide ≥4mg/d v 5-ASA 1.5 to 2.0g/d+BDP 3mg/d	—	—	0.18(0.02, 0.57)	Low**	0.18(0.02, 0.57)	Low §§
Budesonide ⩾4mg/d v Prednisolone 20 to 25mg/d	—	—	1.07(0.55, 1.87)	Low**	1.07(0.55, 1.87)	Low
Budesonide ⩾4mg/d v Prednisolone ⩾30mg/d	—	—	0.53(0.21, 1.13)	Low**	0.53(0.21, 1.13)	Low
Budesonide ⩾4mg/d v Hydrocortisone 100mg/d	—	—	1.84(0.85, 3.85)	Very low**‡‡	1.84(0.85, 3.85)	Very low
Budesonide ⩾4mg/d v Hydrocortisone 356mg/d	—	—	0.87(0.23, 2.24)	Very low**‡‡	0.87(0.23, 2.24)	Very low
Budesonide ⩾4mg/d v Betamethasone 5mg/d	—	—	0.54(0.20, 1.22)	Low**	0.54(0.20, 1.22)	Low
Budesonide ⩾4mg/d v BDP 3mg/d	—	—	0.46(0.24, 0.81)	Low**	0.46(0.24, 0.81)	Low §§
5-ASA 1g/d v 5-ASA 1.5 to 2.0g/d+BDP 3mg/d	—	—	0.35(0.05, 1.08)	Very low**‡‡	0.35(0.05, 1.08)	Very low
5-ASA 1g/d v Prednisolone ≥30mg/d	—	—	1.00(0.42, 2.01)	Moderate ¶	1.00(0.42, 2.01)	Moderate
5-ASA 1g/d v Hydrocortisone 100mg/d	—	—	3.54(1.66, 7.13)	Low**	3.54(1.66, 7.13)	Low §§
5-ASA 1g/d v Betamethasone 5mg/d	—	—	1.03(0.38, 2.21)	Low**	1.03(0.38, 2.21)	Low
5-ASA 1g/d v Budesonide 0.5mg/d	—	—	4.03(1.69, 8.66)	Very low**‡‡	4.03(1.69, 8.66)	Very low §§
5-ASA 1g/d v Budesonide ≥4mg/d	—	—	2.00(1.15, 3.20)	Low**	2.00(1.15, 3.20)	Low §§
5-ASA 1.5 to 2.0g/d v 5-ASA 1.5 to 2.0g/d+BDP 3mg/d	—	—	0.39(0.05, 1.16)	Very low**‡‡	0.39(0.05, 1.16)	Very low
5-ASA 1.5 to 2.0g/d v Prednisolone ≥30mg/d	—	—	1.14(0.48, 2.36)	Moderate ¶	1.14(0.48, 2.36)	Moderate
5-ASA 1.5 to 2.0g/d v Hydrocortisone 100mg/d	—	—	4.01(1.89, 7.83)	Low**	4.01(1.89, 7.83)	Low §§
5-ASA 1.5 to 2.0g/d v Hydrocortisone 356mg/d	—	—	1.85(0.51, 4.45)	Low**	1.85(0.51, 4.45)	Low
5-ASA 1.5 to 2.0g/d v Betamethasone 5mg/d	—	—	1.17(0.43, 2.47)	Very low**‡‡	1.17(0.43, 2.47)	Very low
5-ASA 1.5 to 2.0g/d v Budesonide 0.5mg/d	—	—	4.57(1.85, 9.60)	Low**	4.57(1.85, 9.60)	Low §§
5-ASA 1.5 to 2.0g/d v Budesonide 2 to 2.3mg/d	—	—	2.83(1.76, 4.40)	Low**	2.83(1.76, 4.40)	Low §§
5-ASA 1.5 to 2.0g/d v Budesonide ≥4mg/d	—	—	2.26(1.35, 3.62)	Low**	2.26(1.35, 3.62)	Low §§
5-ASA 4g/d v Prednisolone 20 to 25mg/d	—	—	2.34(1.28, 3.92)	Moderate ¶	2.34(1.28, 3.92)	Moderate §§
5-ASA 4g/d v Prednisolone ≥30mg/d	—	—	1.15(0.49, 2.36)	Moderate ¶	1.15(0.49, 2.36)	Moderate
5-ASA 4g/d v Hydrocortisone 356mg/d	—	—	1.87(0.52, 4.68)	Low**	1.87(0.52, 4.68)	Low
5-ASA 4g/d v Betamethasone 5mg/d	—	—	1.18(0.44, 2.54)	Very low**‡‡	1.18(0.44, 2.54)	Very low
5-ASA 4g/d v Budesonide 0.5mg/d	—	—	4.59(1.90, 9.75)	Very low**‡‡	4.59(1.90, 9.75)	Very low §§
5-ASA 4g/d v Budesonide ≥4mg/d	—	—	2.27(1.35, 3.54)	Low**	2.27(1.35, 3.54)	Low §§

Table S1. Estimates of the effects and quality ratings for the comparison of drugs to induce clinical remission for active distal UC patients. *risk of bias; †Inconsistency; ‡Imprecision; §Indirectness [intransitivity]. ††Cannot be estimated because the drug was not connected in a loop in the evidence network. ¶Contributing direct evidence of moderate quality. **Contributing direct evidence of low or very low quality. ‡‡Indirectness because of the questionable comparability of the trial populations to the target population of NMA or because of intransitivity. §§Greater precision Ratings: High: We have enough confidence in the evidence rating. Moderate: We have moderate confidence in the evidence rating. Low: We have limited confidence in the evidence rating and the true effect might be substantially different from the estimate of the effect. Very low: We have no certainty of confidence in the evidence rating.

Comparison	Direct evidence		Indirect evidence		Network meta-analysis	
	OR(95%CI)	Quality of evidence	OR(95%CrI)	Quality of evidence	OR(95%CrI)	Quality of evidence
**Endoscopic remission**						
Budesonide ⩾4mg/d **v** placebo	2.29(1.42, 3.71)	Low*†	5.81(1.95, 17.27)	Low**	2.55(1.55, 4.12)	Low §§
5-ASA 4g/d v BDP 3mg/d	1.22(0.51, 2.94)	Moderate‡	0.67(0.16, 2.45)	Very low**‡‡	1.27(0.60, 2.51)	Moderate
5-ASA 4g/d v 5-ASA 1.5 to 2.0g/d +BDP 3mg/d	0.60(0.24, 1.46)	Moderate‡	0.29(0.05, 1.61)	Moderate ¶	0.72(0.44, 1.13)	Moderate
BDP 3mg/d v 5-ASA 1.5 to 2.0g/d +BDP 3mg/d	0.55(0.26, 1.17)	Moderate‡	0.51(0.24, 1.09)	Moderate ¶	0.63(0.30, 1.17)	Moderate
5-ASA 1g/d v placebo	6.45(4.23, 9.82)	Low* §	2.47(0.98, 7.59)	Low**	4.97(3.21, 7.51)	Low §§
5-ASA 4g/d v Budesonide 2 to 2.3mg/d	1.19(0.66, 2.16)	Low*‡	1.59(0.65, 3.79)	Very low**‡‡	1.60(0.89, 2.66)	Low
5-ASA 1.5 to 2.0g/d v 5-ASA 1g/d	1.24(0.86, 1.78)	Low* §	0.82(0.33, 2.12)	Low**	1.01(0.64, 1.48)	Low
5-ASA 4g/d v 5-ASA 1.5 to 2.0g/d	1.08(0.73, 1.61)	Very low*§‡	1.16(0.35, 3.78)	Very low**‡‡	1.12(0.68, 1.73)	Very low
5-ASA 1.5 to 2.0g/d v placebo	4.49(2.61, 7.73)	Low*‡	7.72(2.82, 20.13)	Low**	4.89(3.22, 7.16)	Low §§
Budesonide 0.5mg/d **v** placebo	1.36(0.52, 3.56)	Low*‡	1.01(0.34, 3.01)	Low**	1.23(0.39, 2.98)	Low
Budesonide 2 to 2.3mg/d **v** placebo	3.15(1.29, 7.70)	Low*‡	4.94(2.18, 12.04)	Low**	3.53(1.90, 5.95)	Low §§
Budesonide 2 to 2.3mg/d **v** Budesonide 0.5mg/d	2.32(0.99, 5.46)	Low*‡	3.46(1.04, 11.47)	Low**	3.57(1.20, 8.89)	Low §§
Budesonide ⩾4mg/d **v** Budesonide 0.5mg/d	3.42(1.49, 7.86)	Low*‡	1.80(0.58, 5.63)	Low**	2.58(0.91, 5.92)	Low
Budesonide ⩾4mg/d **v** Budesonide 2 to 2.3mg/d	1.47(0.70, 3.11)	Low*‡	0.39(0.12, 1.06)	Very low**‡‡	0.77(0.37, 1.48)	Low
5-ASA 4g/d v placebo	6.86(3.53, 13.34)	Very low*§‡	3.59(1.64, 8.38)	Very low**‡‡	5.36(3.26, 8.38)	Very low §§
5-ASA 4g/d v 5-ASA 1g/d	1.34(0.76, 2.36)	Very low*§‡	0.70(0.32, 1.57)	Very low**‡‡	1.11(0.65, 1.76)	Very low
BDP 3mg/d **v** Prednisolone ⩾30mg/d	1.21(0.60, 2.46)	Moderate‡	2.79(0.56, 15.08)	Low**	1.58(0.68, 3.36)	Moderate
5-ASA 1.5 to 2.0g/d v 5-ASA 1.5 to 2.0g/d +BDP 3mg/d	0.18(0.03, 1.02)	Low*‡	0.78(0.21, 2.78)	Very low**‡‡	0.66(0.44, 0.97)	Low §§
5-ASA 1.5 to 2.0g/d v BDP 3mg/d	0.25(0.04, 1.40)	Low*‡	1.66(0.55, 4.72)	Low**	1.17(0.54, 2.16)	Low
5-ASA 1.5 to 2.0g/d v Prednisolone 20 to 25mg/d	1.47(0.91, 2.38)	Moderate‡	1.30(0.44, 3.80)	Low**	1.40(0.74, 2.34)	Moderate
5-ASA 1g/d v Budesonide 2 to 2.3mg/d	0.95(0.28, 3.20)	Moderate‡	1.63(0.75, 3.63)	Low**	1.50(0.81, 2.59)	Moderate
Budesonide 2 to 2.3mg/d **v** Prednisolone ⩾30mg/d	1.33(0.22, 7.93)	Moderate‡	0.59(0.11, 3.04)	Very low**‡‡	1.20(0.58, 2.22)	Moderate
Budesonide 2 to 2.3mg/d **v** Prednisolone 20 to 25mg/d	0.71(0.22, 2.25)	Very low*†‡	0.97(0.34, 2.92)	Very low**‡‡	1.01(0.49, 1.86)	Very low
5-ASA 1g/d v Prednisolone 20 to 25mg/d	1.36(0.65, 2.86)	Moderate‡	1.30(0.47, 3.42)	Very low**‡‡	1.43(0.74, 2.42)	Moderate
5-ASA 4g/d v Hydrocortisone 100mg/d	11.29(3.02, 42.28)	Low*‡	Not estimable	Not estimable††	3.49(0.91, 9.87)	Low
5-ASA 1.5 to 2.0g/d+BDP 3mg/d v placebo	—	—	17.00(5.21, 41.18)	Low*‡	17.00(5.21, 41.18)	Low §§
Prednisolone 20 to 25mg/d v placebo	—	—	3.76(1.92, 6.76)	Very low**‡‡	3.76(1.92, 6.76)	Very low §§
Prednisolone 20 to 25mg/d v 5-ASA 1.5 to 2.0g/d+BDP 3mg/d	—	—	0.51(0.26, 0.91)	Low*‡	0.51(0.26, 0.91)	Low §§
Prednisolone ⩾30mg/d v placebo	—	—	3.25(1.35, 6.73)	Low*‡	3.25(1.35, 6.73)	Low §§
Prednisolone ≥30mg/d v 5-ASA 1.5 to 2.0g/d+BDP 3mg/d	—	—	0.44(0.18, 0.91)	Very low**‡‡	0.44(0.18, 0.91)	Very low §§
Prednisolone ⩾30mg/d v Prednisolone 20 to 25mg/d	—	—	0.94(0.34, 2.16)	Very low**‡‡	0.94(0.34, 2.16)	Very low
Hydrocortisone 100mg/d v placebo	—	—	2.18(0.47, 6.21)	Low*‡	2.18(0.47, 6.21)	Low
Hydrocortisone 100mg/d v 5-ASA 1.5 to 2.0g/d+BDP 3mg/d	—	—	0.29(0.06, 0.84)	Low*‡	0.29(0.06, 0.84)	Low §§
Hydrocortisone 100mg/d v Prednisolone 20 to 25mg/d	—	—	0.63(0.12, 1.94)	Low*‡	0.63(0.12, 1.94)	Low
Hydrocortisone 100mg/d v Prednisolone ⩾30mg/d	—	—	0.77(0.12, 2.58)	Very low**‡‡	0.77(0.12, 2.58)	Very low
BDP 3mg/d v placebo	—	—	4.66(2.21, 8.67)	Very low**‡‡	4.66(2.21, 8.67)	Very low §§
BDP 3mg/d v Prednisolone 20 to 25mg/d	—	—	1.36(0.51, 2.98)	Very low**‡‡	1.36(0.51, 2.98)	Very low
BDP 3mg/d v Hydrocortisone 100mg/d	—	—	3.18(0.60, 10.50)	Very low**‡‡	3.18(0.60, 10.50)	Very low
Budesonide 0.5mg/d v 5-ASA 1.5 to 2.0g/d+BDP 3mg/d	—	—	0.17(0.05, 0.40)	Moderate ¶	0.17(0.05, 0.40)	Moderate §§
Budesonide 0.5mg/d v Prednisolone 20 to 25mg/d	—	—	0.36(0.09, 1.00)	Low*‡	0.36(0.09, 1.00)	Low §§
Budesonide 0.5mg/d v Prednisolone ⩾30mg/d	—	—	0.43(0.10, 1.06)	Very low**‡‡	0.43(0.10, 1.06)	Very low
Budesonide 0.5mg/d v Hydrocortisone 100mg/d	—	—	0.85(0.13, 3.18)	Very low**‡‡	0.85(0.13, 3.18)	Very low
Budesonide 0.5mg/d v BDP 3mg/d	—	—	0.29(0.08, 0.77)	Low*‡	0.29(0.08, 0.77)	Low §§
Budesonide 2 to 2.3mg/d v 5-ASA 1.5 to 2.0g/d+BDP 3mg/d	—	—	0.48(0.26, 0.81)	Very low**‡‡	0.48(0.26, 0.81)	Very low §§
Budesonide 2 to 2.3mg/d v Hydrocortisone 100mg/d	—	—	2.39(0.54, 7.42)	Very low**‡‡	2.39(0.54, 7.42)	Very low
Budesonide 2 to 2.3mg/d v BDP 3mg/d	—	—	0.83(0.37, 1.64)	Low*‡	0.83(0.37, 1.64)	Low
Budesonide ≥4mg/d v 5-ASA 1.5 to 2.0g/d+BDP 3mg/d	—	—	0.35(0.21, 0.56)	Low*‡	0.35(0.21, 0.56)	Low §§
Budesonide ⩾4mg/d v Prednisolone 20 to 25mg/d	—	—	0.75(0.32, 1.51)	Low*‡	0.75(0.32, 1.51)	Low
Budesonide ⩾4mg/d v Prednisolone ⩾30mg/d	—	—	0.92(0.35, 2.10)	Low*‡	0.92(0.35, 2.10)	Low
Budesonide ⩾4mg/d v Hydrocortisone 100mg/d	—	—	1.77(0.35, 6.08)	Very low**‡‡	1.77(0.35, 6.08)	Very low
Budesonide ⩾4mg/d v BDP 3mg/d	—	—	0.61(0.26, 1.27)	Very low**‡‡	0.61(0.26, 1.27)	Very low
5-ASA 1g/d v 5-ASA 1.5 to 2.0g/d+BDP 3mg/d	—	—	0.67(0.43, 1.02)	Very low**‡‡	0.67(0.43, 1.02)	Very low
5-ASA 1g/d v Prednisolone ≥30mg/d	—	—	1.76(0.71, 3.53)	Moderate ¶	1.76(0.71, 3.53)	Moderate
5-ASA 1g/d v Hydrocortisone 100mg/d	—	—	3.33(0.76, 9.54)	Very low**‡‡	3.33(0.76, 9.54)	Very low
5-ASA 1g/d v BDP 3mg/d	—	—	1.19(0.54, 2.33)	Very low**‡‡	1.19(0.54, 2.33)	Very low
5-ASA 1g/d v Budesonide 0.5mg/d	—	—	5.19(1.55, 12.82)	Very low**‡‡	5.19(1.55, 12.82)	Very low §§
5-ASA 1g/d v Budesonide ≥4mg/d	—	—	2.07(1.00, 3.74)	Low*‡	2.07(1.00, 3.74)	Low §§
5-ASA 1.5 to 2.0g/d v Prednisolone ≥30mg/d	—	—	1.73(0.71, 3.41)	Moderate ¶	1.73(0.71, 3.41)	Moderate
5-ASA 1.5 to 2.0g/d v Hydrocortisone 100mg/d	—	—	3.33(0.75, 10.20)	Very low**‡‡	3.33(0.75, 10.20)	Very low
5-ASA 1.5 to 2.0g/d v Budesonide 0.5mg/d	—	—	5.13(1.49, 12.76)	Very low**‡‡	5.13(1.49, 12.76)	Very low §§
5-ASA 1.5 to 2.0g/d v Budesonide 2 to 2.3mg/d	—	—	1.48(0.76, 2.53)	Low*‡	1.48(0.76, 2.53)	Low
5-ASA 1.5 to 2.0g/d v Budesonide ≥4mg/d	—	—	2.04(1.01, 3.46)	Very low**‡‡	2.04(1.01, 3.46)	Very low §§
5-ASA 4g/d v Prednisolone 20 to 25mg/d	—	—	1.55(0.79, 2.84)	Moderate ¶	1.55(0.79, 2.84)	Moderate
5-ASA 4g/d v Prednisolone ≥30mg/d	—	—	1.89(0.76, 3.94)	Moderate ¶	1.89(0.76, 3.94)	Moderate
5-ASA 4g/d v Budesonide 0.5mg/d	—	—	5.58(1.66, 14.37)	Low*‡	5.58(1.66, 14.37)	Low §§
5-ASA 4g/d v Budesonide ≥4mg/d	—	—	2.22(1.10, 4.11)	Low*‡	2.22(1.10, 4.11)	Low §§

Table S2. Estimates of the effects and quality ratings for the comparison of drugs to induce endoscopic remission for active distal UC patients. *risk of bias; †Inconsistency; ‡Imprecision; §Indirectness [intransitivity]. ††Cannot be estimated because the drug was not connected in a loop in the evidence network. ¶Contributing direct evidence of moderate quality. **Contributing direct evidence of low or very low quality. ‡‡Indirectness because of the questionable comparability of the trial populations to the target population of NMA or because of intransitivity. §§Greater precision Ratings: High: We have enough confidence in the evidence rating. Moderate: We have moderate confidence in the evidence rating. Low: We have limited confidence in the evidence rating and the true effect might be substantially different from the estimate of the effect. Very low: We have no certainty of confidence in the evidence rating.

(A) Clinical remission

5-ASA 4g/d												
0.97 (0.64, 1.33)	5-ASA 1.5 to 2.0g/d											
1.09 (0.69, 1.66)	1.14 (0.76, 1.64)	5-ASA 1g/d										
**1.81 (1.09, 2.81)**	**1.91 (1.15, 3.09)**	1.71 (0.97, 2.79)	Budesonide ⩾4mg/d									
**2.25 (1.42, 3.41)**	**2.38 (1.42, 3.65)**	**2.12 (1.27, 3.36)**	1.28 (0.80, 2.00)	Budesonide 2 to 2.3mg/d								
**3.60 (1.52, 7.36)**	**3.80 (1.59, 7.95)**	**3.40 (1.42, 7.10)**	2.03 (0.91, 4.02)	1.63 (0.72, 3.28)	Budesonide 0.5mg/d							
1.07 (0.57, 1.85)	1.14 (0.60, 2.04)	1.02 (0.48, 1.86)	0.63 (0.28, 1.19)	**0.50 (0.24, 0.93)**	**0.35 (0.11, 0.82)**	BDP 3mg/d						
0.45 (0.05, 1.57)	0.47 (0.05, 1.60)	0.42 (0.05, 1.53)	**0.26 (0.03, 0.95)**	**0.20 (0.03, 0.68)**	**0.14 (0.01, 0.61)**	0.46 (0.05, 1.69)	Betamethasone 5mg/d					
**2.45 (1.02, 4.89)**	**2.60 (1.12, 5.12)**	2.32 (0.96, 4.67)	1.40 (0.62, 2.94)	1.09 (0.55, 1.91)	0.79 (0.24, 1.87)	2.48 (0.85, 5.35)	**11.47 (1.30, 44.04)**	Hydrocortisone 100mg/d				
1.07 (0.46, 2.19)	1.13 (0.48, 2.35)	1.01 (0.40, 2.11)	0.61 (0.25, 1.28)	0.49 (0.20, 1.04)	**0.35 (0.10, 0.88)**	1.01 (0.51, 1.82)	5.25 (0.56, 22.29)	0.50 (0.16, 1.26)	Prednisolone ⩾30mg/d			
**2.32 (1.29, 3.85)**	**2.42 (1.50, 3.70)**	**2.17 (1.26, 3.43)**	1.34 (0.67, 2.41)	1.06 (0.59, 1.77)	0.75 (0.28, 1.74)	2.33 (0.97, 4.58)	**10.92 (1.27, 43.82)**	1.08 (0.42, 2.33)	2.52 (0.90, 5.86)	Prednisolone 20 to 25mg/d		
0.39 (0.06, 1.12)	0.42 (0.06, 1.31)	0.38 (0.06, 1.15)	**0.23 (0.03, 0.69)**	**0.18 (0.03, 0.51)**	**0.13 (0.02, 0.43)**	0.39 (0.06, 1.18)	1.89 (0.12, 8.16)	**0.18 (0.03, 0.62)**	0.42 (0.06, 1.38)	**0.18 (0.03, 0.55)**	5-ASA 1.5 to 2.0g/d +BDP 3mg/d	
**5.04 (3.39, 7.16)**	**5.32 (3.55, 7.74)**	**4.76 (3.03, 7.45)**	**2.88 (1.97, 4.16)**	**2.31 (1.48, 3.54)**	1.61 (0.70, 3.10)	**5.11 (2.48, 9.17)**	**23.90 (2.99, 92.08)**	**2.36 (1.00, 4.76)**	**5.48 (2.30, 11.10)**	**2.31 (1.29, 3.88)**	**21.74 (4.20, 81.17)**	Placebo

(B) Endoscopic remission

5-ASA 4g/d										
1.09 (0.67, 1.69)	5-ASA 1.5 to 2.0g/d									
1.19 (0.67, 1.99)	1.11 (0.71, 1.67)	5-ASA 1g/d								
1.94 (0.90, 3.61)	1.81 (0.92, 3.20)	1.67 (0.84, 2.92)	Budesonide ⩾4mg/d							
1.50 (0.83, 2.47)	1.42 (0.75, 2.36)	1.32 (0.68, 2.21)	0.84 (0.40, 1.62)	Budesonide 2 to 2.3mg/d						
**5.03 (1.60, 12.00)**	**4.69 (1.51, 11.22)**	**4.37 (1.36, 11.23)**	2.71 (0.93, 6.51)	**3.43 (1.15, 8.26)**	Budesonide 0.5mg/d					
1.18 (0.51, 2.44)	1.11 (0.49, 2.24)	1.03 (0.42, 2.08)	0.66 (0.25, 1.40)	0.81 (0.37, 1.57)	**0.30 (0.08, 0.79)**	BDP 3mg/d				
1.75 (0.71, 3.43)	1.65 (0.66, 3.27)	1.53 (0.61, 3.28)	0.98 (0.36, 2.17)	1.18 (0.56, 2.28)	0.44 (0.11, 1.18)	1.59 (0.70, 3.14)	Prednisolone ⩾30mg/d			
1.57 (0.71, 3.02)	1.45 (0.75, 2.39)	1.34 (0.66, 2.36)	0.87 (0.36, 1.81)	1.08 (0.50, 2.07)	**0.39 (0.11, 0.94)**	1.48 (0.55, 3.09)	1.03 (0.35, 2.30)	Prednisolone 20 to 25mg/d		
0.62 (0.36, 1.02)	**0.58 (0.38, 0.87)**	**0.54 (0.33, 0.86)**	**0.34 (0.22, 0.53)**	**0.43 (0.25, 0.74)**	**0.15 (0.05, 0.36)**	0.59 (0.27, 1.14)	**0.41 (0.17, 0.85)**	**0.43 (0.22, 0.82)**	5-ASA 1.5 to 2.0g/d +BDP 3mg/d	
**4.61 (2.67, 7.55)**	**4.30 (2.82, 6.45)**	**3.98 (2.45, 6.39)**	**2.50 (1.60, 3.89)**	**3.20 (1.82, 5.49)**	1.14 (0.38, 2.65)	**4.37 (1.99, 8.44)**	**3.02 (1.24, 6.31)**	**3.21 (1.60, 6.06)**	**15.33 (4.48, 37.95)**	Placebo

(C) Histopathological remission

5-ASA 4g/d						
1.17 (0.45, 2.63)	5-ASA 1.5 to 2.0g/d					
1.62 (0.54, 3.07)	1.42 (0.66, 2.51)	5-ASA 1g/d				
1.77 (0.71, 4.07)	1.71 (0.58, 4.02)	1.26 (0.46, 3.11)	Budesonide 2 to 2.3mg/d			
1.33 (0.13, 4.93)	1.19 (0.12, 4.62)	0.91 (0.09, 3.52)	0.73 (0.10, 2.30)	Prednisolone ⩾30mg/d		
1.72 (0.47, 5.29)	1.49 (0.51, 3.50)	1.15 (0.33, 3.11)	1.01 (0.29, 2.53)	3.05 (0.27, 12.24)	Prednisolone 20 to 25mg/d	
**7.06 (2.02, 16.29)**	**6.32 (2.48, 13.00)**	**4.71 (1.79, 10.28)**	**4.61 (1.18, 11.61)**	**16.83 (1.14, 52.96)**	**5.34 (1.19, 15.25)**	Placebo

(D) Adverse events

5-ASA >3g/d												
0.67 (0.23, 1.47)	5-ASA 1.5 to 3.0g/d											
0.73 (0.22, 1.70)	1.12 (0.49, 2.07)	5-ASA <1.5g/d										
0.50 (0.18, 1.08)	0.87 (0.27, 2.13)	0.83 (0.25, 2.02)	Budesonide >2.5mg/d									
0.64 (0.28, 1.22)	1.10 (0.40, 2.35)	1.07 (0.34, 2.49)	1.40 (0.67, 2.53)	Budesonide 2 to 2.5mg/d								
0.62 (0.17, 1.60)	1.05 (0.24, 2.99)	1.03 (0.22, 3.09)	1.31 (0.41, 2.90)	0.98 (0.31, 2.30)	Budesonide <2mg/d							
0.49 (0.11, 1.25)	0.74 (0.25, 1.66)	0.74 (0.20, 1.91)	1.12 (0.24, 3.45)	0.82 (0.21, 2.21)	1.03 (0.19, 3.25)	BDP 3mg/d						
0.52 (0.07, 1.69)	0.91 (0.10, 3.38)	0.89 (0.10, 3.34)	1.17 (0.14, 3.61)	0.84 (0.13, 2.78)	1.09 (0.12, 4.03)	1.50 (0.12, 5.82)	Betamethasone 5mg/d					
0.47 (0.12, 1.20)	0.82 (0.19, 2.43)	0.80 (0.16, 2.33)	1.02 (0.29, 2.51)	0.74 (0.28, 1.77)	0.95 (0.21, 2.63)	1.35 (0.21, 4.14)	1.63 (0.18, 6.30)	Hydrocortisone 100mg/d				
0.66 (0.10, 1.98)	1.04 (0.22, 3.00)	1.00 (0.18, 2.90)	1.41 (0.26, 4.48)	1.04 (0.21, 3.20)	1.35 (0.18, 4.70)	1.47 (0.36, 3.96)	2.27 (0.17, 10.14)	1.76 (0.25, 6.96)	Prednisolone >25mg/d			
1.24 (0.33, 3.43)	1.86 (0.81, 3.88)	1.80 (0.72, 3.95)	2.82 (0.66, 8.37)	2.12 (0.51, 6.13)	2.67 (0.48, 8.68)	3.23 (0.84, 8.40)	4.76 (0.43, 20.09)	3.83 (0.66, 12.08)	2.98 (0.54, 9.85)	Prednisolone 20 to 25mg/d		
0.44 (0.05, 1.74)	0.66 (0.11, 2.25)	0.69 (0.09, 2.58)	1.05 (0.13, 4.15)	0.80 (0.10, 2.91)	0.96 (0.09, 4.10)	1.04 (0.15, 3.82)	1.65 (0.08, 8.46)	1.29 (0.10, 5.55)	0.97 (0.10, 3.66)	0.42 (0.05, 1.56)	5-ASA 1.5 to 3.0g/d +BDP 3mg/d	
0.70 (0.30, 1.49)	1.20 (0.43, 2.66)	1.17 (0.38, 2.88)	1.51 (0.74, 2.61)	1.16 (0.58, 2.25)	1.42 (0.47, 3.32)	2.01 (0.46, 5.93)	2.54 (0.38, 9.01)	1.97 (0.52, 4.82)	1.75 (0.35, 5.99)	0.73 (0.19, 1.99)	3.40 (0.38, 11.73)	Placebo

Table S3. Sensitivity analysis of outcomes by excluding trials with a high risk of bias. The efficacy was estimated in the triangle, comparing column-defining with row-defining treatments. The estimates of effects were summarized as odds ratios (ORs) with their corresponding 95% credible intervals (CrIs) respectively. For the efficacy assessment, ORs greater than 1 favor the column-defining treatment, while for adverse effects, ORs greater than 1 favor the row-defining treatment. Results with significant statistical differences are shown in bold. 5-ASA, 5-aminosalicylic acid; BDP, beclomethasone dipropionate.
